# Mutation rate plasticity in rifampicin resistance depends on *Escherichia coli* cell–cell interactions

**DOI:** 10.1038/ncomms4742

**Published:** 2014-04-29

**Authors:** Rok Krašovec, Roman V. Belavkin, John A. D. Aston, Alastair Channon, Elizabeth Aston, Bharat M. Rash, Manikandan Kadirvel, Sarah Forbes, Christopher G. Knight

**Affiliations:** 1 Faculty of Life Sciences, The University of Manchester, Michael Smith Building, Manchester M13 9PT, UK; 2 School of Science and Technology, Middlesex University, London NW4 4BT, UK; 3 Statistical Laboratory, DPMMS, University of Cambridge, Cambridge CB3 0WB, UK; 4 Department of Statistics, University of Warwick, Warwick CV4 7AL, UK; 5 Research Institute for the Environment, Physical Sciences and Applied Mathematics, Keele University, Keele ST5 5BG, UK; 6 Wolfson Molecular Imaging Centre, The University of Manchester, Manchester M20 3LJ, UK; 7 Manchester Pharmacy School, The University of Manchester, Manchester M13 9PT, UK

## Abstract

Variation of mutation rate at a particular site in a particular genotype, in other words mutation rate plasticity (MRP), can be caused by stress or ageing. However, mutation rate control by other factors is less well characterized. Here we show that in wild-type *Escherichia coli* (K-12 and B strains), the mutation rate to rifampicin resistance is plastic and inversely related to population density: lowering density can increase mutation rates at least threefold. This MRP is genetically switchable, dependent on the quorum-sensing gene *luxS*—specifically its role in the activated methyl cycle—and is socially mediated via cell–cell interactions. Although we identify an inverse association of mutation rate with fitness under some circumstances, we find no functional link with stress-induced mutagenesis. Our experimental manipulation of mutation rates via the social environment raises the possibility that such manipulation occurs in nature and could be exploited medically.

## Abstract (web summary)

The factors varying mutation rate at a particular site in a single genotype remain elusive. Here, Krašovec *et al*. show that mutation rates at sites conferring resistance to rifampicin in *Escherichia coli* decrease with population density, and that mutation-rate plasticity is controlled by the *luxS* gene.

## Graphical abstract

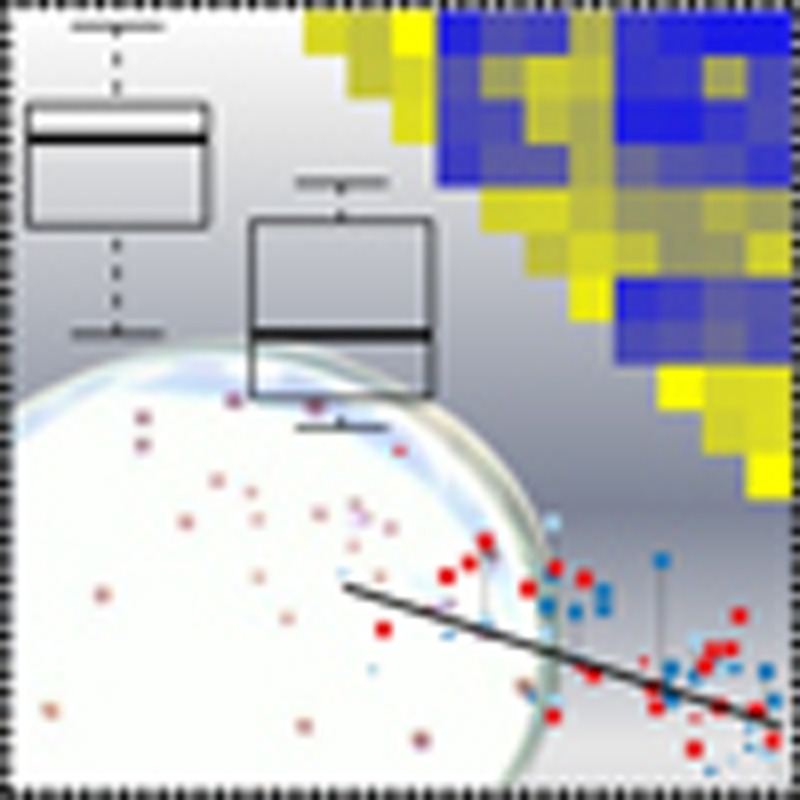

Mutation rate has long been appreciated as a fundamental factor in evolutionary genetics^1^,^2^. In nature, mutation rates are typically minimized, as far as population genetic constraints allow^3^. However, rates of spontaneous mutation can vary both between^3^,^4^ and locally within^5^,^6^ genotypes. In particular systems, such as the model bacterium *E. coli*, there is abundant variation in mutation rates among natural isolates^7^. For a single genotype, the question of whether and how mutation rates at any particular site might vary (mutation rate plasticity, MRP) is of particular interest^8^. Several theoretical works have shown that increasing mutation rate, specifically when an organism is displaced from an adaptive peak, may be advantageous^9^,^10^,^11^. Indeed, in evolutionary computing, such MRP is used to optimize performance^12^. Evidence for MRP in nature comes from experiments showing that the number of mutations can increase during environmental^13^ or genetic stress^14^. However, in such cases, many potential causes of MRP are confounded. This includes direct mutagenic effects of the environment and any physiological responses, adaptive or otherwise, affecting mutation rate.

Here, using *E. coli*, we identify plasticity in the rate of mutation to rifampicin resistance. We find this MRP to be mediated by the population density, to be genetically switchable, dependent on the quorum-sensing gene *luxS* and to act via cell–cell signalling. This link between mutation rate and a social system opens up a new area in which bacteria may manipulate each other and, potentially, humans may manipulate bacteria. The relationship with stress-induced mutagenesis is considered and mutation rate control mechanisms potentially involved are discussed.

## Results

### Decreasing mutation rate with increasing absolute fitness

**Figure 1:**
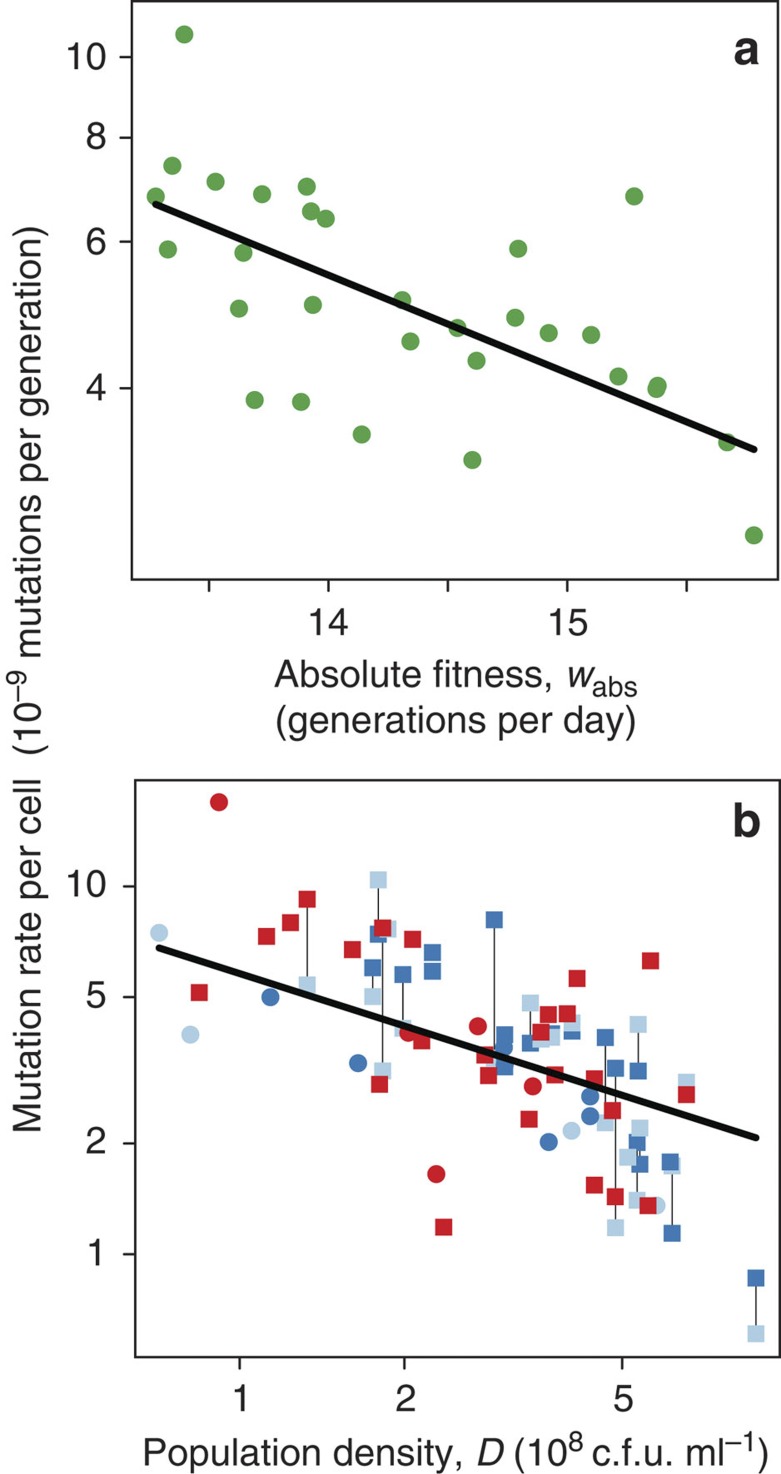
MRP in *E. coli* strains. Relationship of mutation rate (μ) per cell (**a**) to absolute fitness (*w*_abs_) in wild-type *E. coli* K-12, and (**b**) to final population density (*D*) in *E. coli* B strains. In **a**, the line is the fitted curve (log_2_(*μ*)=7.9−0.39 × *w*_abs_) from Model 1 (see Methods). In **b**, dark and light blue indicate, respectively, the Ara^−^ (REL606) and Ara^+^ (REL607) ancestral B strains, and red indicates the strain evolved for 20,000 generations (REL8593A). Circles are monocultures, squares are cocultures; thin lines link estimates from two strains in the same coculture. The line is the fitted curve (log_2_(*μ*)=15−4.7 × log_2_(*D*)) from Model 2. Note that mutation rate and population density axes are logarithmic.

To explore variation in mutation rate within a single genotype, we used *E. coli* K-12 cells in classical fluctuation assays^15^. These assays identify mutational events in the *rpoB* gene by counting cells resistant to the antibiotic rifampicin (Rif^R^) arising in the absence of rifampicin (non-selective environment). Different amounts of nutrient were provided (50–1000, mg l^−1^ of glucose), allowing cells to achieve different numbers of generations per day (that is, different absolute fitness, *w*_abs_). We find variation of mutation rate related to changing *w*_abs_ (Fig. 1a): mutation rate doubles with every reduction in *w*_abs_ by 2.6 (1.9–3.9, 95% confidence interval (CI)) generations per day (testing slope by analysis of variance (ANOVA): *N*=30, *F*_1,28_=35, *P*=2.4 × 10^−6^; Model 1 in Methods).

### MRP mediated by population density

In Fig. 1a, the independent variable, *w*_abs_, is affected by many parameters, including culture volume, inoculum size, viability, productivity and nutrient availability^16^. In addition, simply modifying available nutrients confounds different potentially causal effects, such as changes in the time spent in different phases of the culture cycle. It is therefore unclear which factor or factors are determining the observed MRP. To identify such factors, we sought to manipulate several of them independently of each other within the same experiment. Specifically, we tested four non-mutually exclusive hypotheses about this MRP it is (i) a direct response to nutrient (glucose) concentration; (ii) an effect intrinsic to the strain (for example, cellular ageing or different cumulative effects of stress because of different amounts of time or numbers of divisions in different phases of the culture cycle); (iii) related to population density; or (iv) related to the competitiveness of the biological environment. These hypotheses each make different predictions; specifically, under each hypothesis, respectively, we expect mutation rate to relate to (i) glucose concentration ([*glc*]), (ii) the number of cell divisions, (iii) final population density (*D*), or (iv) relative fitness (*w*_rel_). As before, we assayed mutation rates to Rif^R^, except this time we used cocultures of two strains. This allowed us to test strains with different fitnesses in the same environment at the same time. To accomplish this, we used *E. coli* B strains either ancestral or evolved in minimal glucose medium for 20,000 generations^17^. We conducted experiments in which conditions were varied by manipulating [*glc*] (50–1,500 mg l^−1^), the strains paired (ancestral or evolved), culture volume (1, 1.5, 10 and 15 ml) and culture period (~24 or 45 h). We fitted a linear model for mutation rate containing each of the factors testing hypotheses (i–iv) above and their interactions, sequentially removing non-significant effects. We found that the only significant effect on mutation rate among those tested was final population density (*D*) alone (Fig. 1b): a reduction in *D* of 77% (61–96%, 95% CI) gives a doubling in mutation rate (testing slope by ANOVA: *N*=80, *F*_1,43_=14, *P*=6.0 × 10^−4^; Model 2 in Methods; see Supplementary Figs 1–3 for equivalent plots of the other three factors and Supplementary Note 1 for tests of modelling assumptions). In other words, we find strong evidence for an effect of final population density on mutation rate (hypothesis (iii) above), but no such evidence supporting alternative hypotheses (numbers (i), (ii) and (iv) above).

### MRP dependent on *luxS*

**Figure 2:**
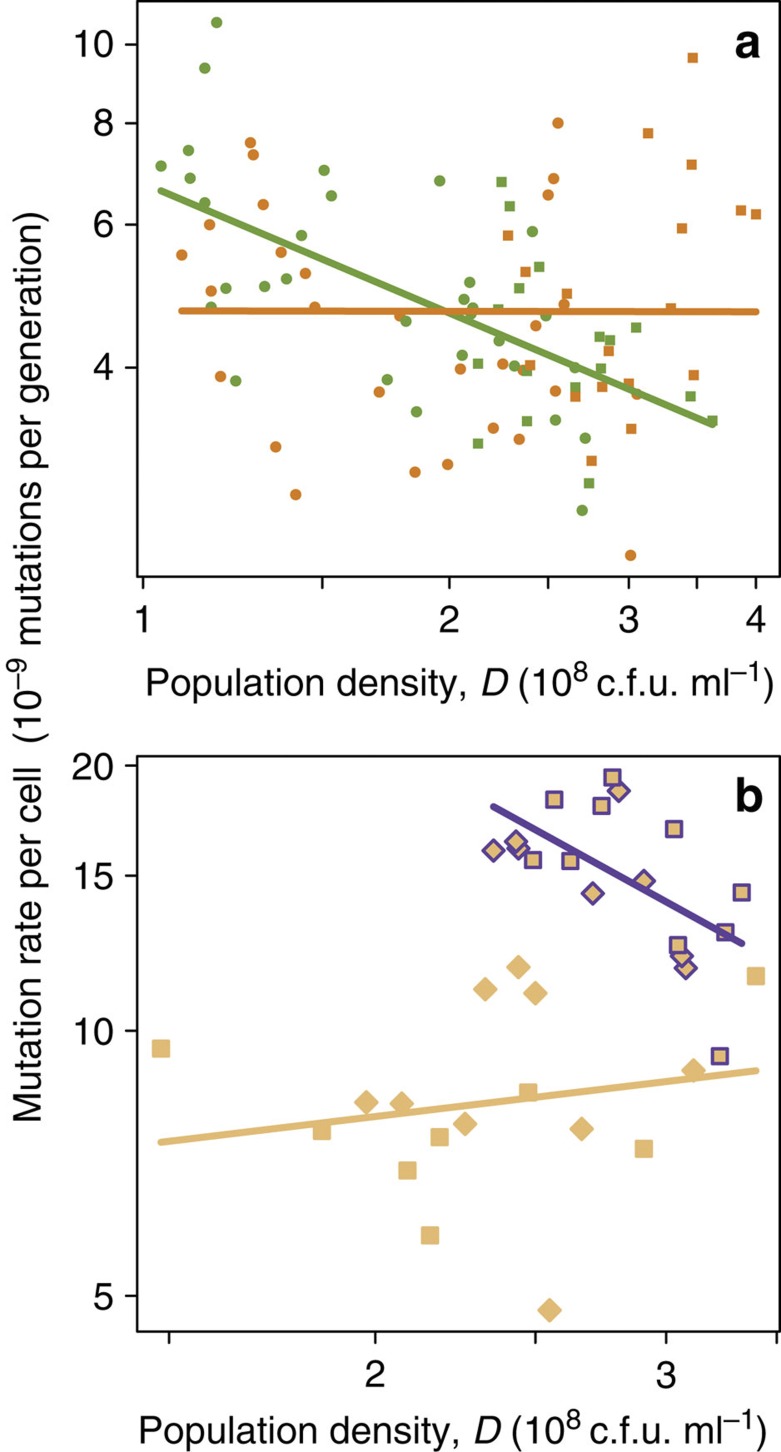
Role of *luxS* gene in MRP. (**a**) Relationship of mutation rate and population density (*D*) in wild-type *E. coli* K-12 (green) and otherwise isogenic Δ*luxS* mutant (KX1228; orange). Lines are the fits from Model 3 (see Methods). Note that some data is common with Fig. 1a. (**b**) Mutation rate in *E. coli* Δ*luxS* mutant (KX1200) (cocultured with either wild-type (diamonds) or Δ*luxS* mutant (KX1228; squares)) cells in either aspartate-containing (outlined) or minimal (no outline) media. Lines are the fits from Model 6. Note the different logarithmic scales used in each plot; also note that the density axes are shorter than in Fig. 1b, as K-12 strains do not grow to as high density in this medium as B strains. In **a**, the range of densities considered is extended by including cocultures with B strains (squares), although these do not behave significantly differently to monocultures (Model 3).

The identified inverse relationship between mutation rate and population density opens the question of the underlying molecular mechanism. Quorum-sensing mediated by *luxS* is responsible for a variety of density-dependent behaviours in bacteria^18^. We therefore hypothesized that the identified MRP is dependent on the *luxS* gene. To test the role of *luxS,* we estimated mutation rate to Rif^R^at different densities in an *E. coli* K-12 Δ*luxS* mutant both alone and in coculture with the ancestral B Ara^−^ strain. The Δ*luxS* mutant grows to approximately the same density as the K-12 wild-type (for example, 2.4±0.09 × 10^8^ ml^−1^ and 2.3±0.09 × 10^8^ ml^−1^, respectively, in minimal medium with 250 mg l^−1^ glucose; mean±s.e., *N*=12 in each case) and has, on average, a mutation rate that is not significantly different (testing strain effect by ANOVA: *N*=95, *F*_1_,_91_=0.25, *P*=0.62; Model 3 in Methods). However, we observed a significant difference in the slope of mutation rate in response to *D* between strains (Fig. 2a, testing interaction effect by ANOVA: *N*=95, *F*_1,91_=12, *P*=7.3 × 10^−4^; Model 3 in Methods). Furthermore, the Δ*luxS* mutant shows a response (slope) not significantly different from zero (strain contrast test: *N*=95, *t*_91_=0.015, *P*=0.99; Model 3 in Methods), showing that the identified MRP is not only density dependent but also *luxS* dependent. The same conclusion may be reached by analysing cocultures alone (Model 4 in Methods; Supplementary Fig. 4).

### LuxS-dependent MRP via the activated methyl cycle

*luxS* encodes an enzyme, part of the activated methyl cycle^19^, that splits *S*-ribosyl homocysteine (HCY) to give HCY and 4,5-dihydroxy-2,3-pentanedione (DPD). This reaction is the unique metabolic route to DPD that forms the quorum-sensing signal autoinducer 2 (AI-2). HCY is not a unique product of this reaction, but is depleted in the Δ*luxS* mutant^19^. HCY is a precursor of various molecules including the structurally unidentified signalling molecule autoinducer 3 (AI-3)^20^. We hypothesized that if MRP is mediated by AI-2 quorum-sensing, then the Δ*luxS* mutant will be functionally complemented by adding synthetic DPD. Alternatively, if MRP is mediated by other metabolic effects of *luxS* deletion, then these effects will be functionally complemented by adding aspartate to the medium, which is metabolized to give HCY^20^. Adding synthetic DPD (Supplementary Fig. 5 and Model 5 in Methods) to low density cultures of either wild-type or Δ*luxS* mutant, at a range of concentrations shown to be physiologically active, gives no evidence for a reduction in either the wild-type or Δ*luxS* strain’s mutation rate (Supplementary Fig. 6). In fact, the mutation rate shows a slight but significant change in the opposite direction, increasing with added DPD (testing slope by ANOVA: *N*=62, *F*_1,36_=22, *P*=4.0 × 10^−5^; Model 6 in Methods). In contrast, supplying aspartate to the medium at a level known to complement metabolic defects of the Δ*luxS* mutant restores the density dependence to levels seen in the wild-type: a reduction of 49% (33–89%, 95% CI) in density doubles the mutation rate (Fig. 2b; testing the interaction between aspartate and density dependence by ANOVA: *N*=35, *F*_1,31_=8.1, *P*=0.0079; Model 7 in Methods). We therefore conclude that it is the metabolic product of LuxS, not the known signalling molecule AI-2, that affects the mutation rate.

This finding, that density-dependent (Fig. 1b) and *luxS*-dependent (Fig. 2a) mutation rate is mediated by the activated methyl cycle, not the AI-2 signalling, is consistent with the fact that we see similar MRP in both the K-12 (Figs 1a and 2) and B lineages (Fig. 1b) of *E. coli*. Unlike K-12 strains, B strains lack much of the *lsr* operon (all except *lsrB, lsrF* and *lsrG*) used to detect and process AI-2 (ref. 21). We observe similar independence from the *lsr* operon in the K-12 lineage where a Δ*lsrK* mutant, lacking the kinase required for AI-2 uptake and processing, shows MRP indistinguishable from that of the K-12 wild-type (Supplementary Fig. 7; testing for difference between genotypes by likelihood ratio (LR): *N*=126, LR_10,7_=3.3, *P*=0.34).

### MRP via cell–cell interactions

**Figure 3:**
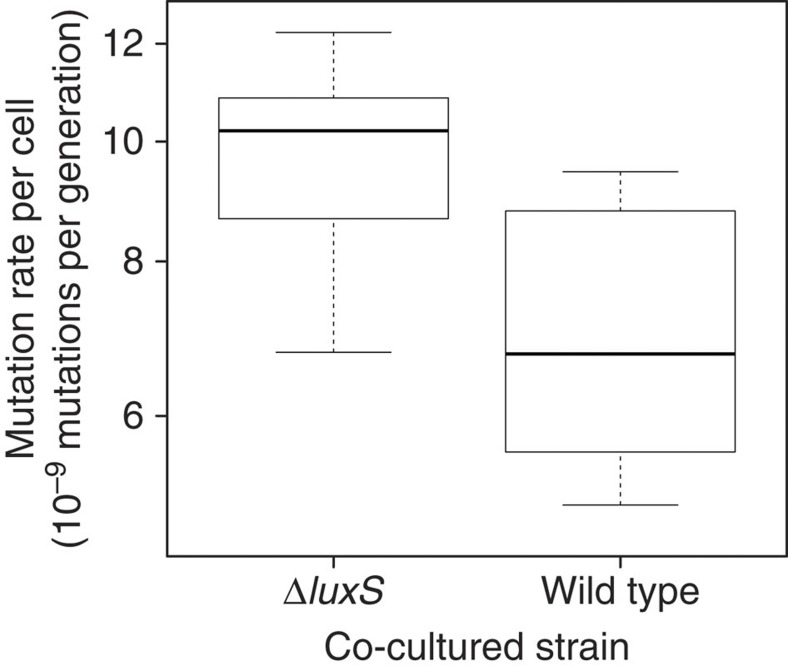
Role of social context in MRP. Mutation rate to Rif^R^ in *E. coli* K-12 (KX1102) dependent on cocultured strain: either wild-type or Δ*luxS* mutant (KX1228). Heavy bars are median values, boxes indicate the interquartile range, and whiskers indicate the maximum and minimum values recorded. Mutation rate in KX1102 is significantly different depending on the cocultured strain (*N*=19, *F*_1,17_=12, *P=*0.0034; Model 8), but not on overall culture density (Supplementary Fig. 8; Model 8). Note the logarithmic scale on the mutation rate axis.

The observed wild-type MRP depends on the biological environment (Figs 1b and 2a), suggesting action via cell–cell interactions. Yet counter-intuitively, it is the non-secreted product of LuxS that is required for MRP. In addition, the effect of the *luxS* deletion on mutation rate is not functionally complemented by coculture with the wild-type (Fig. 2b and Model 7 in Methods). To test explicitly whether the effect of the Δ*luxS* mutation acts via cell–cell interactions, we cocultured K-12 cells marked via chloramphenicol (Cm) resistance either with wild-type K-12 or Δ*luxS* mutants (both sensitive to Cm). We then measured mutation rates to Rif^R^ in the marked fraction of the population. We find that populations comprising either combination of strains grow to very similar final population densities (*D*=2.4±0.097 × 10^8^ and 2.3±0.17 × 10^8^ for wild-type and Δ*luxS* mutant, respectively, in minimal medium with 250 mg l^−1^ glucose; mean±s.e., *N*=9 and 10, respectively). However, mutation rate to Rif^R^ depends strongly on the identity of the cocultured strain, not on overall population density *D* (Supplementary Fig. 8): average mutation rate increases by over a third in the presence of the Δ*luxS* mutant relative to the presence of wild-type cells (Fig. 3; testing effect of biological environment by ANOVA: *N*=19, *F*_1,17_=12, *P=*0.0034; Model 8 in Methods). As in Fig. 2b, this effect of the Δ*luxS* mutant may be functionally complemented by aspartate in the medium, with an increase in average mutation rate (Supplementary Fig. 9, testing effect of aspartate on average mutation rate by ANOVA: *N*=72, *F*_1,64_= 50, *P* =1.4 × 10^−9^; Model 9 in Methods). This confirms that the action of *luxS* is both via cell–cell interactions and via the activated methyl cycle. Taken together, it is clear that the MRP we observe is dependent upon the social environment provided by *luxS*-dependent processes.

### Hypothesis generation via transcription analysis

In stress-induced mutagenesis, increases in mutation rate occur predominantly via induction of error-prone DNA polymerases IV and V (PolIV and PolV; encoded by the *dinB* and *umuC/D* genes, respectively)^22^. We therefore assayed transcription of these genes in the wild-type and Δ*luxS* mutant at high density, where we see a minimized mutation rate in the wild-type, but not in the Δ*luxS* strain (Fig. 2a). We detect expression of both *dinB* and *umuC* at similar levels in the two genotypes (Supplementary Fig. 10, where we also find that, as expected, *luxS*-dependent expression of *lsr* genes does differ between genotypes). The small differences that we do see are marginally non-significant in the opposite direction to that predicted if error-prone DNA polymerases were responsible for the observed modulation in mutation rate (i.e., greater *dinB* and *umuC* expression in the wild-type than Δ*luxS* mutant: strain contrast test *N*=48, *t*_41_=2.0, *P*=0.056; Model 10 in Methods).

**Figure 4:**
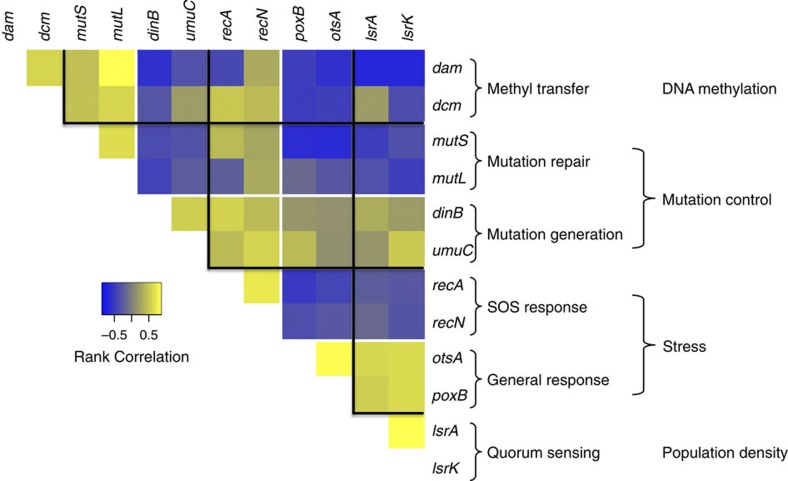
Transcription correlations in published *E. coli* studies. Selected downstream effectors associated with population density, stress, mutation control and DNA methylation are analysed. Spearman rank correlations between the expression of two genes are shown by colour. Each value is a weighted median across 96 separate studies. Associated *P*-values and partial correlations (controlling for the correlations among groups of genes) are given in Supplementary Fig. 11.

Such negative results are potentially misleading, because we cannot look comprehensively across the culture cycle, associated environmental differences and relevant genotypes. Therefore, to generate hypotheses about potential mechanisms involved in the observed MRP, we looked more widely across growth conditions/phases, genetic and environmental manipulations by analysing published expression data^23^. As a proxy for density-dependent effects, we considered expression of *lsr* genes that are transcribed in response to AI-2. We focused on the correlation of density-dependent expression with representative downstream effectors of stress responses, mutation control and DNA methylation (all directly or indirectly linked with mutational processes). We identify strong positive correlations of density-dependent expression with the general stress response and mutation generation (error-prone polymerase genes). At the same time, we identify strong negative correlations of density-dependent expression with the SOS stress response, mutation repair (*mut* DNA mismatch repair genes) and methyl transferases (Fig. 4). It is possible that these strong correlations among several groups of genes may mask correlations between specific gene groups. We therefore controlled for correlations with other groups of genes using partial correlations (Supplementary Fig. 11). When controlled in this way, the signs of the correlations of density-dependent expression with the SOS stress response and DNA mismatch repair become less consistent (notably for *mutS*). However, the signs of the observed correlations of density-dependent expression with the general stress response, error-prone DNA polymerases and methyl transferases are maintained and in some cases strengthened by using partial correlations (notably for *dcm*; Supplementary Fig. 11).

## Discussion

Much of the interest in MRP concerns its potential evolutionary consequences^8^. We do not yet know whether an adaptive explanation is appropriate for the MRP identified here. Theory shows that such relationships may be adaptive when mutation rate is inversely related to fitness^9^,^10^,^11^; the precise nature of the relationship that maximizes the expected rate of fitness increase is mathematically derivable under some circumstances^24^. In such models, it is only when a population is close to an adaptive peak that minimizing mutation rate is beneficial, so when a genotype is displaced from an adaptive peak, the deleterious effects of a raised mutation rate are outweighed by the potential for increasing fitness via beneficial mutations. Fitness is the important variable theoretically, whereas we find that cell density is the important variable *in vivo*. These variables will be correlated only under some circumstances. Thus, we see fitness-dependent MRP only in some cases (for example, a relationship in Fig. 1a, but not in Supplementary Fig. 2).

Nonetheless, the direction of MRP we see in response to cell density is both consistent with adaptive theory and biologically remarkable. The paradigm of stress-induced mutagenesis^8^ relates the induction of error-prone DNA polymerases (PolIV and PolV)^22^ and the downregulation of repair proteins^25^ to the key stationary-phase stress factor RpoS. RpoS is expressed only late in the culture cycle and therefore at relatively high densities. On its own, this would, intuitively, lead to a positive relationship between cell density and mutation rate, the opposite of what we find (we are aware of one other study involving an inverse relationship between population density and the frequency of reversion in *Salmonella typhimurium* Thy^−^ mutants^26^). This intuition is largely confirmed by our analysis of published *E. coli* expression data (Fig. 4 and Supplementary Fig. 11). As expected, stress responses, particularly the general stress response (members of the RpoS regulon), are typically positively correlated with PolIV and PolV gene expression and negatively correlated with *mut* gene expression. However, both the general stress response and expression of error-prone DNA polymerases are positively correlated with the density-induced expression in this data; the opposite of what would be expected if this MRP were explained by stress-induced mutagenesis. This is consistent with the effect of aspartate that we identify (see Supplementary Note 2) and existing work demonstrating that, in glucose minimal media as used here, RpoS expression is not functionally related to population density^27^. Therefore, while we cannot rule out the possibility that some aspects of mechanism may be shared with RpoS or SOS stress responses, or that relationships may be identified via a more nuanced interpretation of gene regulation or what constitutes ‘stress’, the MRP observed here does not appear to be functionally related to stress.

Our data suggest hypotheses for the mutagenic mechanism(s) underlying the MRP identified. For instance, the inverse relationship between population density and *mutS* expression^28^ (see also Fig. 4) may be caused primarily by the correlations of each with stress responses rather than any specific relationship between the two (Supplementary Fig. 11). Modulation of *mutS* expression, independent of stress, could therefore be involved in the minimization of mutation rate that we see at high densities (Figs 1b and 2a). Another hypothesis comes from our finding that it is the metabolic role of *luxS* that is involved in the modulation of mutation rate. This metabolic role is in the activated methyl cycle, which is named because of its importance in mediating the availability of activated methyl groups in the cell. These methyl groups are used, for instance, in DNA methylation, something that has long been known to affect mutation rates at particular sites^29^. Specifically, adenine residues in GATC sites are methylated by Dam methylase^30^ and these are mutational hot spots^31^. It is notable that two of the key positions in the *rpoB* gene, where mutation results in Rif^R^ in wild-type *E. coli* K-12, as assayed here, are the adenines in a GATC site (position 1,714 on the positive and 1,715 on the negative strand)^32^. It is therefore reasonable to hypothesize that the modulation in mutation rate we observe could be effected by variation in the methylation of GATC mutational hot spots by Dam methyl transferase. This would also be consistent with the expression of the *dam* gene, which is inversely associated with density-dependent expression (Fig. 4 and Supplementary Fig. 11). Targets for the Dcm methylase are also mutational hot spots^29^ and *dcm* expression is also inversely correlated with density-dependent expression (Supplementary Fig. 11). However, sites resulting in Rif^R^ do not include nucleotides methylated by Dcm, hence, even if there is MRP involving such sites, we were not able to observe it in our assays. Also, *E. coli* B strains, which display MRP (Fig. 1b), are lacking in Dcm activity^33^. We therefore believe that Dam-mediated rather than Dcm-mediated modulation of mutational hot spots provides the most plausible hypothetical mechanism for the MRP identified here. The testing of such hypotheses and elucidation of relevant pathways is an important target for future work.

The mechanism of MRP we have identified is dependent on *luxS*, which in turn is involved in communication within and between diverse microbial species^18^. It has recently been suggested that polymorphism in *E. coli* of genes downstream of *luxS*, such as *lsrK* considered here, is maintained by a process of social evolution^21^. In this scenario, strains lacking such genes (for example, *E. coli* B used here) are social cheats. The fact that we find mutation rate to be under the control of *luxS*-mediated cell–cell interactions adds a new dimension to such social processes. Finally, as many virulence factors are under quorum-sensing control^34^, quorum-sensing is a current target for anti-virulence drugs^35^. When we remove the *luxS*-dependent quorum-sensing of some cells in a mixed coculture, we boost the emergence of *de novo* antibiotic resistance in other cells (Fig. 3). Such a boost could be a significant side effect if it also occurred in response to quorum-sensing inhibitors. Equally, we have shown that this mechanism of density-dependent MRP is independent of the best-characterized *E. coli* quorum-sensing signalling system (*lsrK*-dependent AI-2 signalling). We therefore speculate that enhancing this alternative cell–cell interaction (as we do in Figs 1, 2, 3 by increasing the density of interacting cells) may be a route to slow the pervasive emergence of microbial antibiotic resistance^36^, thereby improving the efficacy of antibiotic treatment.

## Methods

### Strains

*E. coli* K-12 strains KX1102 (*luxS*^+^*lsrK*^+^ Ara^+^ Δ*lacZYA*::Cm), KX1200(Δ*luxS*::Cm *lsrK*^+^ Ara^+^) and KX1228 (Δ*luxSlsrK*^+^ Ara^+^) were derived from the wild-type K-12MG1665 (*luxS*^+^*lsrK*^+^ Ara^+^)^37^. The *lsrK*::Cm deletion in the parent of KX1448 (*luxS*^+^Δ*lsrK* Ara^+^) was constructed by Karina Xavier using the red swap protocol described by Datsenko and Wanner^38^. To eliminate the Cm-resistance cassette, the FLP recombinase expressing plasmid pCP20 was introduced in the parent yielding KX1448 (ref. 38). *E. coli* B strain REL606 (*luxS*^+^Δ*lsrK*Ara^−^) is the ancestor of all B strains used in this study. REL607 (*luxS*^+^ Δ*lsrK*Ara^+^) is a spontaneous Ara^+^ revertant from REL606 (ref. 17). REL8593A Ara-1 (*luxS*^+^Δ*lsrK* Ara^−^) was derived from REL606 after 20,000 generations of batch culture in a glucose-limited environment^17^,^39^. During experimental evolution the fitness of REL8593A increased by ~70% relative to REL606 and REL607 via several beneficial mutations^39^. REL8593A retains the ancestral mutation rate^40^. Strains in cocultures are distinguished by a visible arabinose (Ara) marker or Cm-resistance marker. B and K-12 strains used in this study possess an *rpoB* gene with an identical DNA sequence and it is located on the same position within the genome.

### Media

We used Milli-Q water for all media. Tetrazolium arabinose agar (TA) and Davis minimal medium (DM) were prepared according to Lenski *et al*.^17^ (on TA agar Ara^−^ strains are red, and Ara^+^ strains are white or pinkish). Magnesium, thiamine, carbon source (3 g l^−1^ L-arabinose or various concentrations of D-glucose), tetrazolium red (Sigma T8877) and 0.5 mM aspartate dipeptide (BACHEM) were sterile filtered and added to a cooled medium as necessary. Selective TA medium is TA supplemented with freshly prepared antibiotic 50 μg ml^−1^ rifampicin. For KX1102, selective TA was supplemented with both 50 μg ml^−1^ of rifampicin (Rif) and 25 μg ml^−1^ of Cm. For all cell dilutions, sterile saline (8.5 g l^−1^ NaCl) was used. Media were solidified as necessary with 15 g l^−1^ of agar (Difco).

### Fluctuation tests

We used fluctuation tests designed by Luria and Delbrück^15^. Specifically, strains were first inoculated from frozen stock and grown in 10 ml liquid LB medium at 37 °C (shaken at 120 r.p.m.) to OD_600_ =~1 (~7 h). As a preconditioning step, each strain was transferred (via a 2,000-fold dilution) to 10 ml of non-selective liquid DM medium supplemented with a particular concentration (80–1,500 mg l^−1^) of glucose and allowed to grow overnight at 37 °C (120 r.p.m.). Cells were again diluted into fresh medium giving *N*_0_ (the initial number of viable cells, containing no rifampicin resistant, Rif^R^, mutants) of ~7,000. The same medium was used as in the preconditioning step. Where *N*_0_ included two strains, they were distinguished by alternative Ara- or Cm-resistance markers. Three volumes of cultures were used: 1 and 1.5 ml cultures were grown in 96 deep-well plates, and 10 ml cultures in glass universal tubes. Cultures were then grown to saturation (24–28 h at 37 °C at 250 r.p.m.). To minimize spatial effects, we positioned each independent culture on the plate randomly. The final number of viable cells, *N*_*t*_, was determined by plating an appropriate dilution on solid non-selective TA medium. *N*_*t*_ was calculated with 3–6 cultures per mutation rate estimate. Evaporation (routinely monitored by weighing plate before and after incubation) was accounted for in the *N*_*t*_ value. For 1 ml cultures, this was on average 12% of the population density, calculated per millilitre of the medium. We obtained the observed number of Rif^R^ mutants, *r*, by plating the entirety of remaining cultures (at least 12 per estimate) onto solid selective TA medium that allows spontaneous Rif^R^ mutants to produce colonies. Plates were incubated at 37 °C and mutants were counted at the earliest possible time after plating. For Rif plates, this was 44–48 h, when both Rif and Cm were used the incubation time was 68–72 h.

For Figs 1a,b and 2a,b and Fig. 3 we used 10, 18, 13, 7 and 6 independent experimental blocks, respectively. Across Fig. 1a,b, the number of plates per estimation is at least 12 (median=17, interquartile range 13–21), across Fig. 2a,b is at least 17 (median=21, interquartile range 20–21) and across Fig. 3 is at least 21 (median=21, interquartile range 21–23).

### Estimation of mutation rates

For calculating the number of mutational events *m,* we used the Ma–Sandri–Sarkar maximum-likelihood method^41^,^42^. This method is valid over the entire range of values of *m*^43^,^44^ and is implemented by the FALCOR web tool^45^ that uses Stewart’s Equation 1 to calculate s.d. of *m*^46^. The mutation rate per cell per generation, *μ*, is calculated as *m* divided by the number of cells at risk, *N*_*t*_. Only values of *m* >0.3 were considered to be valid^44^ and were analysed further.

### Fitness assay

In two-strain fluctuation tests, the neutral Ara marker or Cm resistance allowed us to assess the initial and final number of viable cells (*N*_0_ and *N*_*t*_ respectively) of the two strains. From these values we calculated each strain's realized Malthusian parameter log(*N*_*t*_*/N*_0_). Relative fitness (*w*_rel_) was then calculated as the ratio of the realized Malthusian parameters^17^, averaged across 3–6 replicates. When we used one strain in a fluctuation test *w*_rel_ was designated as 1. Absolute fitness (*w*_abs_) was measured as number of generations (*G*) per 24 h, calculated as *G*=log_2_(*N*_*t*_/*N*_0_)/*t,* where *t* is time in days.

### *In vitro* synthesis of AI-2

*In vitro* synthesis of (S)-DPD (AI-2) was carried out as previously described^47^. We supplemented DM medium with 1, 10, 100, 400 and 1,000 μM of synthetic AI-2.

### Bioluminescence assay

Standard bioluminescence assay was performed according to Surette and Bassler^48^. Bioluminescence were integrated across a 16-h culture of *Vibrio harveyi* BB170, ATCC number BAA-1117, at 30 °C with aeration in AB medium, with the given concentration of DPD. Overnight cultures were diluted to an OD_600_ of 0.2 and then further diluted to 1:5,000 in fresh AB medium. Cultures (180 μl) were then aliquotted in a 96-well plate. Bioluminescence was recorded every 30 min using a Biotek Synergy-2 luminometer.

### Quantitative real-time PCR

Primers were designed using the tool available at Invitrogen (http://tools.lifetechnologies.com/content.cfm?pageid=9716) to give a product between 70 and 200 bp. Primer sequences are the following: *lsrB* forward (F): (5′-CCCAGTGTTTCTGGTCAGGT-3′) and reverse (R): (5′-AACCGCAGAAACGATAATGG-3′), *lsrK* F:(5′-TCGACACCTATACGCTGCTG-3′) and R:(5′-CGCAGGTGATACCAGGTTTT-3′), *dinB* F:(5′-ACGCCTACAAAGAAGCCTCA-3′) and R:(5′-TTGCAGCTCGTTGAAGATTG-3′), *umuC* F:(5′-TGGGGGATTTCTTCAGTCAG-3′) and R:(5′-TTCCTCTGCCCTCTTTAGCA-3′). The duration of the reverse transcription reaction was 60 min at 45 °C, the reaction was stopped at 95 °C for 15 min. Reverse transcription products were subjected to 50 cycles of PCR amplification (1 min at 95 °C for denaturation, 1 min at 62 °C for annealing and 30 s at 72 °C for extension). At the end, we run a dissociation curve by gradually increasing temperature from 55 to 95 °C (0.2 °C per second). The iScript One-Step RT-PCR Kit with SYBR Green was used. All reactions were performed with Bio-Rad (M J Research) Chromo4 real-time PCR machine, and we used Opticon Monitor 3 for analysis.

### Analysis of published expression data

Data were taken from the Colombos transcription database version 2 (20131118), containing 131 different studies covering a wide range of environmental and genetic perturbations in *E. coli*^23^. For all combinations of genes of interest, rank correlations of expression and associated *P* values were calculated across samples within a single study. In each case, the median value of the correlation and *P* value was calculated across studies, weighted by –log_10_(*P*) (that is, a weighting from 0 to 16, the limit of numerical accuracy, in favour of studies where strong correlations were found or that were powerful enough to find weaker correlations). We included all studies where there was sufficient data to calculate both full and partial correlations. For partial correlations this requires that, when controlling for correlations with *N* other genes, there are at least *N*+3 samples with data for all *N*+2 genes. Ninety-six studies met these criteria for all genes of interest and were included. Only two example of genes of interest were taken for each subgroup, as increasing the number of genes reduces the number of studies in which there are sufficient data available to calculate correlations. However, similar results are found using different representative genes.

### Statistical analysis

All statistical models were fitted using the nlme package in R^49^. This enabled the inclusion within the same model of experimental factors (fixed effects), blocking effects (random effects) and factors affecting variance (giving heteroscedasticity). Note that many of the models are heteroscedastic and accounting for this involves fitting one or more parameters. Therefore, the *P* values used in model simplification (comparing two models one with and one without an effect of interest, where heteroscedasticity parameter(s) may be fitted differently in each case) will not be identical to the *P* values given in the ANOVA tables for effects within a single model (Supplementary Tables 1–10). To see how we tested model assumptions, see Supplementary Note 1, Supplementary Figs 12 and 13 and Supplementary Table 11. Box–Cox power transformations^50^ of mutation rate in models with mutation rate as the response consistently gave a maximum likelihood for a power (*λ*) significantly <1 (untransformed mutation rate), and not significantly different from zero (log-transformed mutation rate); see Supplementary Fig. 14. Therefore, log_2_-transformed mutation rate was considered in all the models below. The same was true of modelling bioluminescence (Model 5 below). Details of models and their fitting are given below and diagnostic plots in Supplementary Figs 15–24. ANOVA tables for each model are given in Supplementary Tables 1–10. Where relevant in those tables, the level of a factor is given in parentheses next to an effect (for example, ‘Intercept (wild-type)’ implies that the intercept is the value for the wild-type, and a subsequent ‘Strain’ effect will be the difference of another strain considered from that).

### Model 1

The model giving the fitted line in Fig. 1a is the log_2_ mutation rate as a function of absolute fitness (*w*_abs_). A random effect of experimental block explained only a tiny proportion of the variance (6.0 × 10^−8^) and was, therefore, not included in the final model (the same applies to each of the models below). Various experimental effects could in principle affect the variability of results (heteroscedasticity), specifically: experimental blocks again and their order, the number of plates used to estimate the final population size and the number of mutation events (*m*), the estimated value and coefficient of variation of *m*, the estimated density of the culture (*D*) and fitted value of the mutation rate, the estimated inoculum size, the proportion of the culture remaining following evaporation and glucose concentration ([*glc*] treated as either a continuous or discrete variable). Models including each of these effects were fitted and compared, plus models containing combinations of effects that individually improved the model. The best model (lowest Akaike information criterion, AIC) was achieved allowing the variance to change as [number of plates used to estimate the final population size]^–1.6^. See Supplementary Table 1, Fig. 1a and Supplementary Fig. 15.

### Model 2

Glucose concentration ([*glc*]), absolute fitness (*w*_abs_), final culture density (log_2_(*D*)) and relative fitness (*w*_rel_), plus all interactions, were considered as explanatory variables for mutation rate measured for strains in cocultures including both ancestral and evolved strains (20,000 generations in minimal glucose). To minimize any issues with error in these explanatory variables (Supplementary Note 1), the median (for example, *D*_med_) of each of these values was used within each strain–environment combination (where environment includes nutrient, competitor strain, culture volume and growth period; for *D*_med_ there were 2 (median) 1.25–3 (interquartile range) measurements for each of 30 unique strain–environment combinations). There is also potential for pseudoreplication in the cases where mutation rate estimates for both strains in a culture were available (Fig. 1b). This was accounted for by including a random effect of culture (nested within experimental block) in the model (giving s.d.=0.56 and 0.45 at block and culture level respectively, with residual s.d.=81). This model was simplified, sequentially removing non-significant effects not required in higher-level interactions until any further removal resulted in a significantly worse model (LR test *P*<0.05, that is, finding the minimal adequate model). Heteroscedasticity relating to experimental variables was tested for as above, including the effect of strain (either the strain for which the mutation estimate was made or the cocultured strain, either separating the alternatively marked versions of the ancestral B strain or not) and competition time (in hours). The resulting model contained only the effect of final culture density (log_2_(*D*)), with variance increasing as [number of plates used for estimating the number of mutational events]^–2.0^. See Supplementary Table 2, Fig. 1b and Supplementary Fig. 16.

### Model 3

Mutation rate was considered as a response to strain (wild-type K-12 or Δ*luxS*), cocultured strain (in monocultures this was the strain itself and in cocultures the wild-type B strain REL606) and density (log_2_(*D*), centred on the average density: log_2_(*D*)_centred_) and their interactions. Testing the experimental variables as above, significant heteroscedasticity among strains was identified in the final model with the Δ*luxS* mutant strain having 1.4 times the variance of the other strains and variance increasing as [number of plates used to estimate the final population size]^–1.0^. This model was simplified by sequentially removing non-significant effects not required in higher-level interactions until any further removal resulted in a significantly worse model (LR test *P*<0.05, that is, finding the minimal adequate model). The resulting model contained no effect of cocultured strain, only the effect of strain, final culture density (log_2_(*D*)_centred_) and their interaction. See Supplementary Table 3, Fig. 2a and Supplementary Fig. 17.

### Model 4

The difference in mutation rate between two strains in coculture was considered as function of final population density (log_2_(*D*), centred on the average density: log_2_(*D*)_centred_), strain pairing (wild-type K-12, Δ*luxS* or ancestral B Ara^+^, each paired with ancestral B Ara^−^) and their interaction. Heteroscedasticity associated with experimental variables was tested for as above, although where appropriate, variables were tested for each cocultured strain separately (either the ‘winning’ or ‘losing’ strain) and together (for example, when asking whether the number of mutational events estimated had an effect on variance, the numbers for each strain separately and the total number of events for both strains were all tested). Variance was found to increase with [fitted value]^1.3^ and to be 2.7-fold greater in competitions where the B Ara^+^ strain was out-competed. The fitted lines for K-12 wild-type and ancestral B Ara^+^ were very similar, giving non-significant treatment contrasts between them (*P*=0.11 for both the main effect and interaction with density) and with higher *P* values than contrasts between other pairs of strains. Therefore, these two strains were combined, which improved the model (lower AIC, LR_7,9_=4.4, *P*=0.11). See Supplementary Table 4 and Supplementary Figs 4 and 18.

### Model 5

log_2_(Bioluminescence), where Bioluminescence is integrated over the course of the experiment, was considered as a response to the concentration of DPD added ([DPD]), the batch of DPD used and their interaction. The interaction was non-significant (LR_8,7_=2.3, *P*=0.13) but both main effects were significant. Heteroscedasticity relating to experimental variables was tested for as above, variance increasing significantly as [fitted value]^42^ and with decreasing DPD concentration (relative variance of 1 for 6.25 μM DPD, 0.2 for concentrations of 12.5–50 μM and 0.066 for 100 μM). See Supplementary Table 5, and Supplementary Figs 5 and 19.

### Model 6

Mutation rate was considered as a response to strain (wild-type (K-12) or Δ*luxS*), DPD concentration and their interaction. As the shape of any DPD concentration response was unknown, power transformation was used (Box–Cox as above), which gave a maximum likelihood for a transformation close to logarithmic (*λ*=0.099). This model was simplified, sequentially removing non-significant effects (not required in the interaction) until any further removal resulted in a significantly worse model (LR test *P*<0.05; that is, finding the minimal adequate model). Heteroscedasticity associated with experimental variables was tested for as above. Variance was found change as [number of plates used to estimate the mutation rate]^−2.6^. The resulting model contained only the effect of DPD concentration. See Supplementary Table 6 and Supplementary Fig. 20.

### Model 7

Mutation rate in the Δ*luxS* CM-marked mutant was considered as a response to culture density (*D*), competitor (wild-type K-12 or Δ*luxS*), aspartate (presence/absence) and all possible interactions. This model was simplified, sequentially removing non-significant effects not required in higher-level interactions until any further removal resulted in a significantly worse model (LR test *P*<0.05, that is, finding the minimal adequate model). Heteroscedasticity relating to experimental variables was tested for as above, variance increasing significantly as [inoculum size]^–1.7^ in the final model. The final model contained only the effect of final culture density (*D*), the effect of aspartate and their interaction. See Supplementary Table 7, Fig. 2b and Supplementary Fig. 21.

### Model 8

The final culture density (log_2_(*D*)), the identity of the cocultured strain (wild-type or the Δ*luxS* mutant) and their interaction were considered as explanatory variables for mutation rate measured in the Cm-marked wild-type strain. This model was simplified, sequentially removing non-significant effects, not required in the interaction, until any further removal resulted in a significantly worse model (LR test *P*<0.05, that is, finding the minimal adequate model). Heteroscedasticity relating to experimental variables was tested for as above. The resulting model contained only the effect of cocultured strain, allowing the variance to increase as [coefficient of variation of *m*]^0.81^. See Fig. 3, Supplementary Table 8 and Supplementary Figs 8 and 22.

### Model 9

Mutation rate was considered in response to strain (CM-marked wild-type or Δ*luxS* mutant), competitor (wild-type (K-12) or Δ*luxS*) and aspartate (presence/absence) and all possible interactions. Removal of the three-way interaction made the model significantly worse (LR_10,9_=4.3 *P*=0.037 despite its marginally non-significant *P* value by ANOVA, see Supplementary Table 8, AIC was also lower for the complete model than any simplification of it), therefore no simplification was possible. Heteroscedasticity relating to experimental variables was tested for as above, variance increasing as [*m*]^0.25^. See Supplementary Table 9, and Supplementary Figs 9 and 23.

### Model 10

Expression (measured as *C*_t_, the time taken in minutes to reach a threshold of amplification) was considered as a response to the particular genes assayed (*dinB, umuC, lsrB* and *lsrK*), the strain (wild-type or Δ*luxS*) and their interaction, including an effect of experimental block. Both the interaction and the experimental block effect were significant (*P*<0.05 comparing a reduced model to the full model). However, the results for *dinB* and *umuC* were very similar; considering these genes together improved the model (lower AIC, LR_11,13_=3.6 *P*=0.17). Heteroscedasticity relating to experimental variables was tested for as above, variance increasing significantly as [fitted value]^2.7^. Variance also differed among strains (relative variance for wild-type=1, for Δ*luxS*=1.7) and experimental blocks (block A=1 and block B=2.5) See Supplementary Table 10, and Supplementary Figs 10 and 24.

The data and R code used to construct Figs 1, 2, 3 and their associated models are available as Supplementary Data set 1 and Supplementary Methods, respectively.

## Author contributions

R.K. and C.G.K. designed and analysed the experiments, carried out by R.K. All authors contributed to scientific direction of the project. M.K. synthesized and S.F. tested DPD, B.M.R. carried out quantitative real-time PCR. The paper was written by R.K. and C.G.K. and incorporates all other authors’ comments.

## Additional information

**How to cite this article:** Krašovec, R. *et al*. Mutation rate plasticity in rifampicin resistance depends on *Escherichia coli* cell–cell interactions. *Nat. Commun.* 5:3742 doi: 10.1038/ncomms4742 (2014).

## Supplementary Material

Supplementary Information and Supplementary Data Supplementary Figures 1-24, Supplementary Tables 1-11, Supplementary Notes 1-2, Supplementary Methods and Supplementary References

Supplementary Data 1 KrasovecData.xlsx contains the data used in Figs. 1-3. With the R code in the Supplementary Methods, it may be used to reconstruct these figures and their associated analyses
