# Supplementary material for: Mutation rate plasticity in rifampicin resistance depends on Escherichia coli cell–cell interactions
Source: Nat Commun. 2014 Apr 29;5:3742. doi: 10.1038/ncomms4742 (PMC4007418; doi:10.1038/ncomms4742)
Supplement: Supplementary Information and Supplementary Data — Supplementary Figures 1-24, Supplementary Tables 1-11, Supplementary Notes 1-2, Supplementary Methods and Supplementary References [file ncomms4742-s1.pdf]

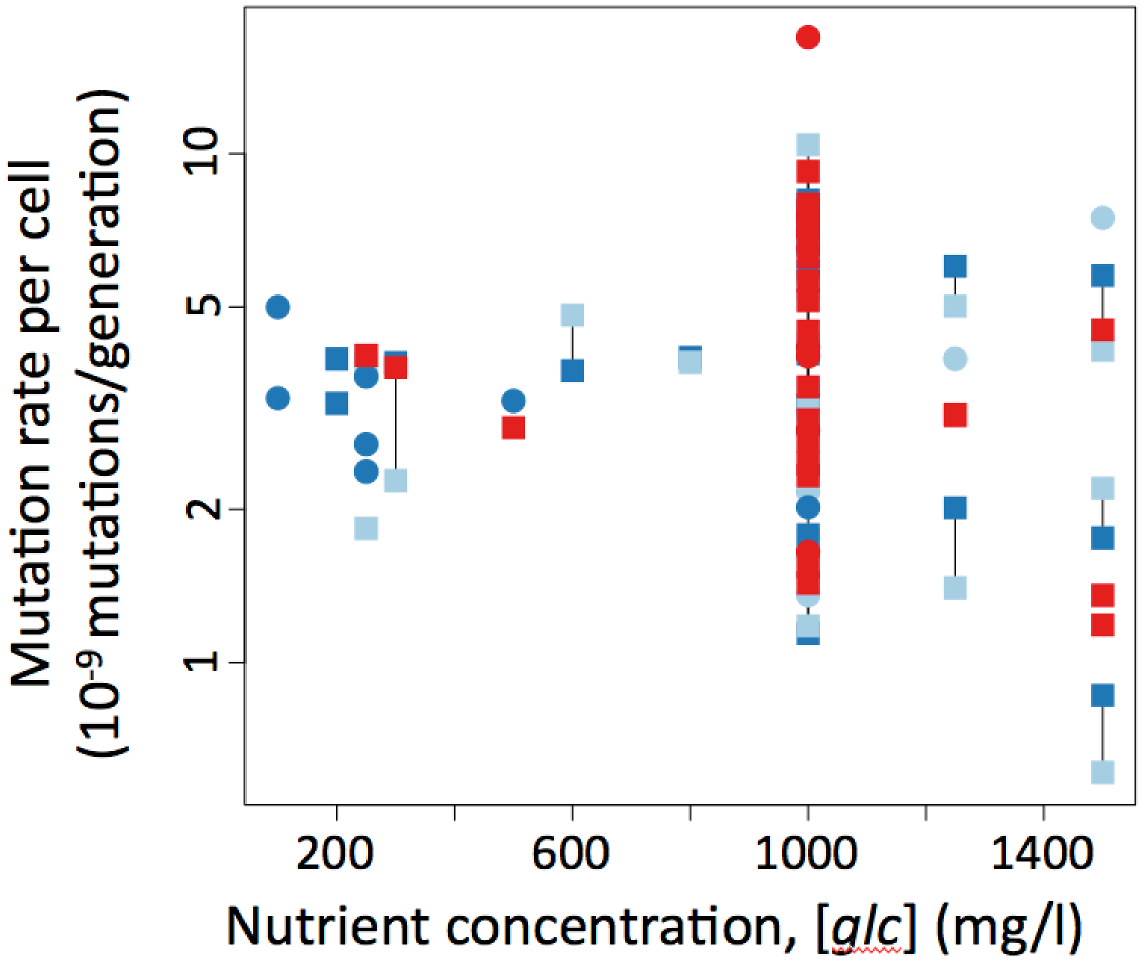

Supplementary Figure 1 | Mutation rate to rifampicin resistance in relationship to glucose concentration ([*glc*]) in *E. coli* B strains. Dark and light blue indicate respectively the Ara$^-$ (REL606) and Ara$^+$ (REL607) ancestral B strains, red indicates the strain evolved for 20,000 generations (REL8593A). Circles are monocultures, squares are co-cultures; thin lines link estimates from two strains in the same co-culture. Note that mutation rate axis is logarithmic.

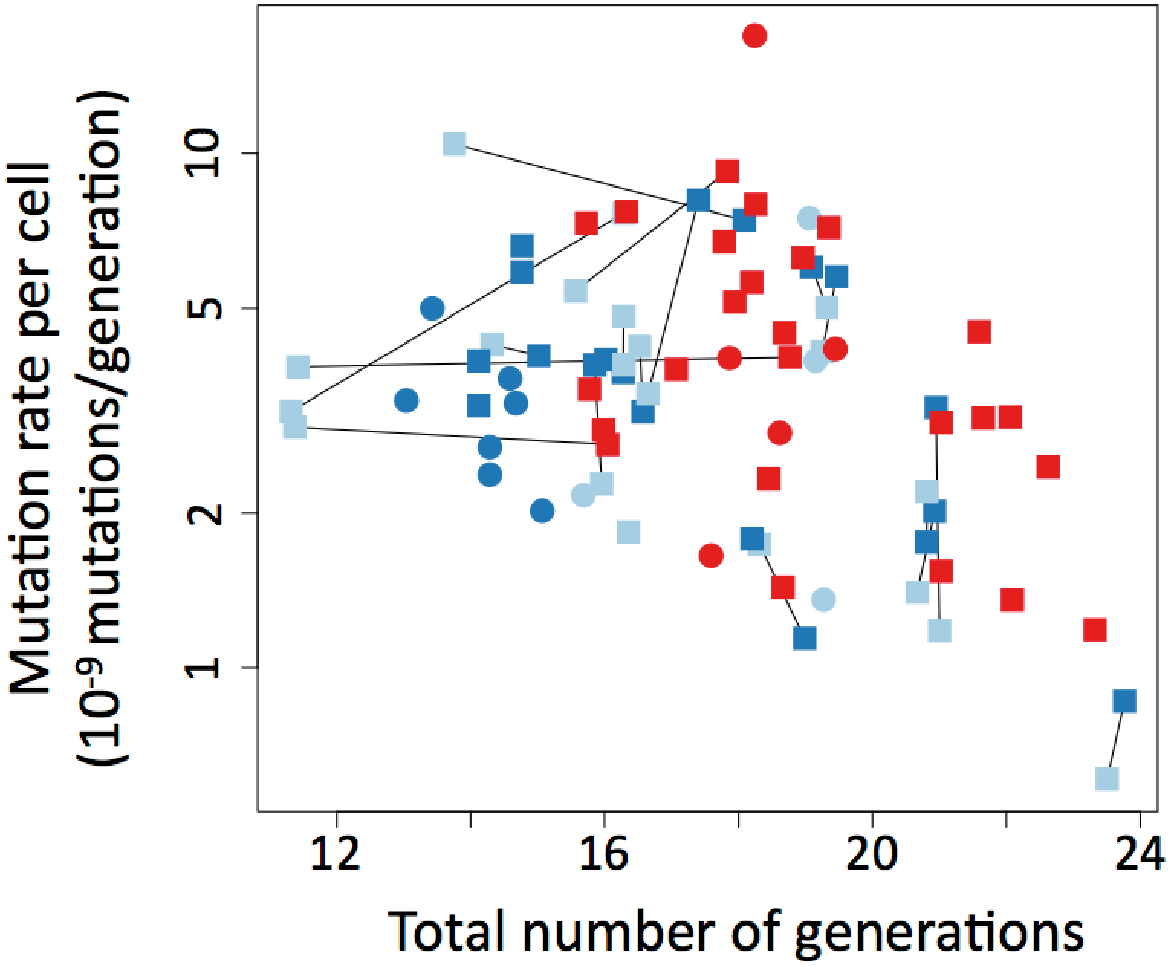

Supplementary Figure 2 | Mutation rate to rifampicin resistance in relationship to the number of generations achieved by *E. coli* B strains. Dark and light blue indicate respectively the Ara^- (REL606) and Ara^+ (REL607) ancestral B strains, red indicates the strain evolved for 20,000 generations (REL8593A). Circles are monocultures, squares are co-cultures; thin lines link estimates from two strains in the same co-culture. Note that mutation rate axis is logarithmic.

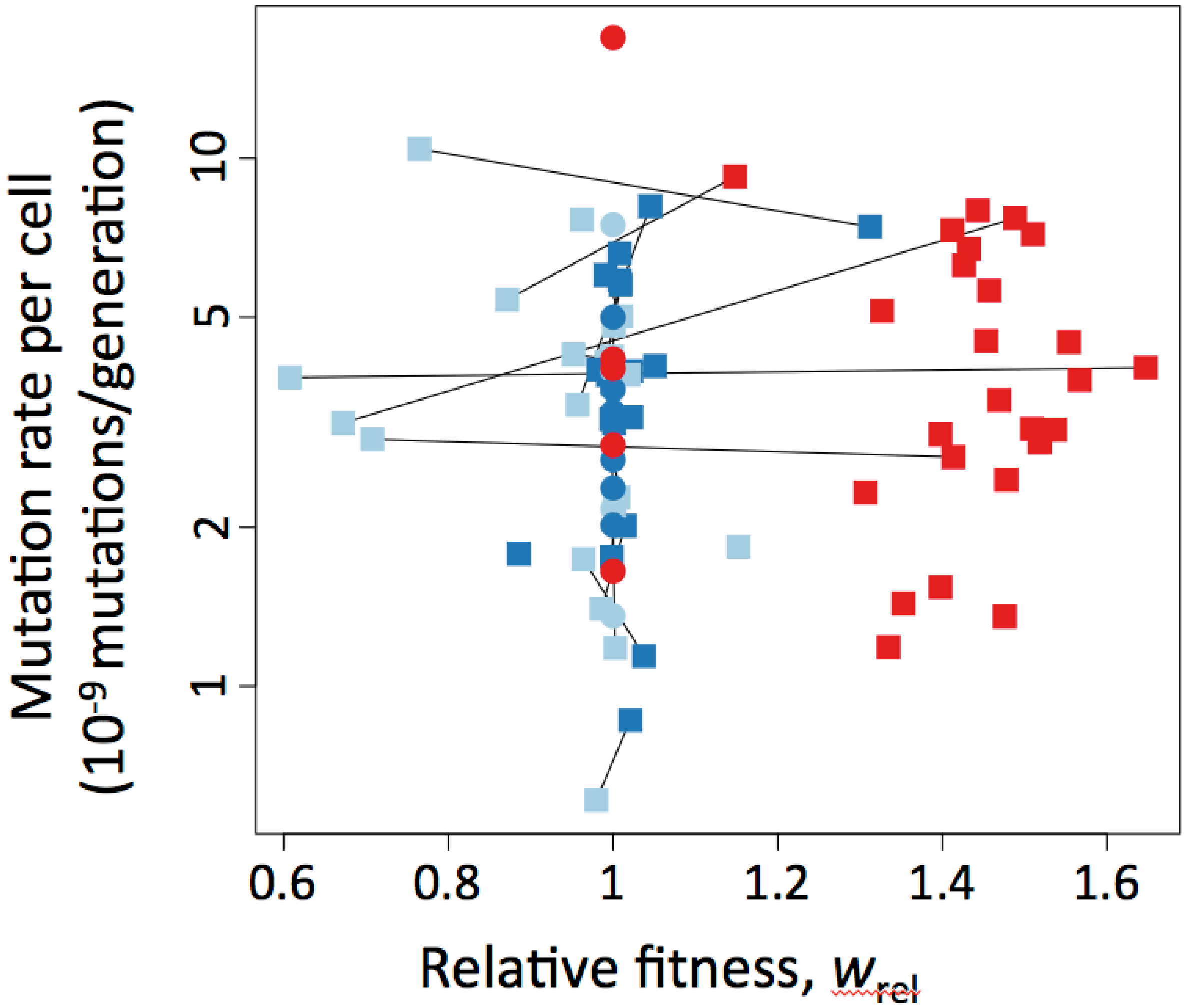

Supplementary Figure 3 | Mutation rate to rifampicin resistance in relationship to relative fitness (w_{rel}) in *E. coli* B strains. Dark and light blue indicate respectively the Ara^- (REL606) and Ara^+ (REL607) ancestral B strains, red indicates the strain evolved for 20,000 generations (REL8593A). Circles are monocultures, squares are co-cultures; thin lines link estimates from two strains in the same co-culture. Note that mutation rate axis is logarithmic.

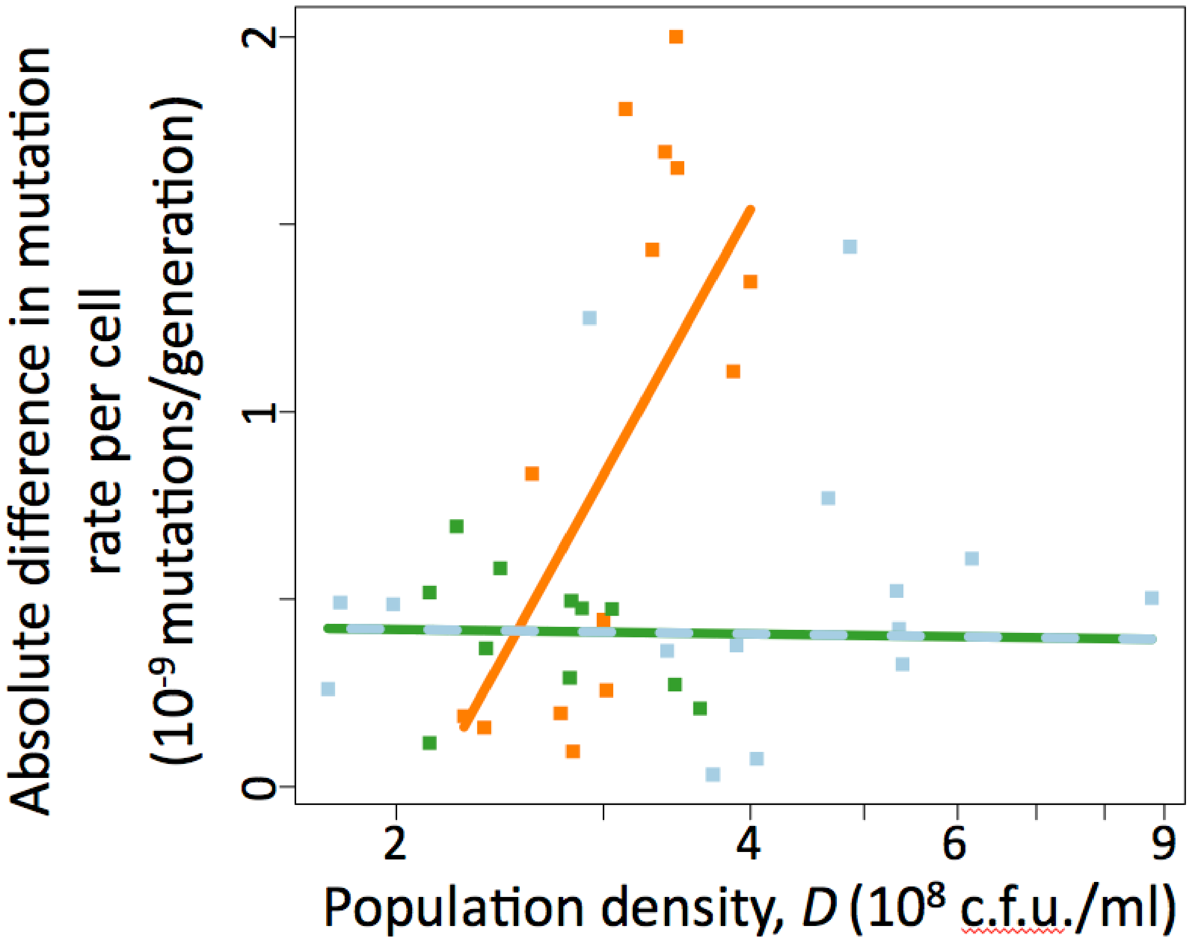

Supplementary Figure 4 | Difference between mutation rate to rifampicin resistance for two *E. coli* strains in co-culture in relationship to the final density of cells (*D*). A wild-type B strain (ancestral B Ara^-) was co-cultured with an alternatively marked version of the same strain (ancestral B Ara^+, pale blue), a wild-type K-12 strain (green) or a Δ*luxS* K-12 strain (Orange). Line colours correspond to strain colours and are fits from Model 4 (see Methods). The pairings have significantly different responses to *D* ($N = 40$, $F_{1,36} = 20$, $P = 6.7 \times 10^{-5}$). Qualitatively the same result is found analysing only the pairs containing a K-12 strain ($N = 25$, $F_{1,21} = 23$, $P = 1.1 \times 10^{-4}$ for the same comparison of slope among pairings)

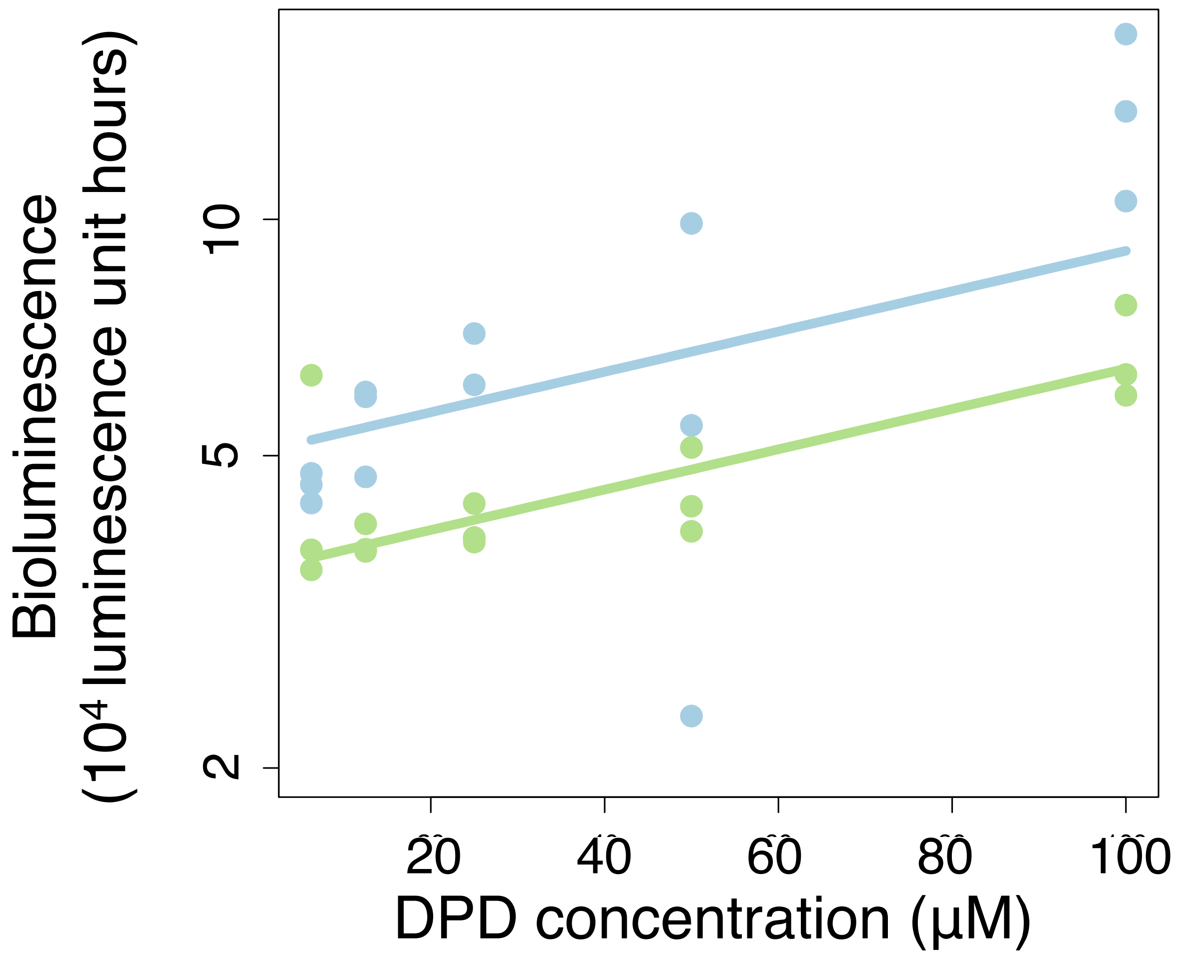

Supplementary Figure 5 | Efficacy of synthetic DPD: Bioluminescent response of *Vibrio harveyi* BB170 cultures to added DPD. Biolumiescence is integrated over the course of a 16-hour experiment and plotted against concentration of DPD added (DPD batches are considered separately: batch A blue and batch B green). The lines are fits from Model 9 (see Methods), with details in Supplementary Table S9 and Supplementary Fig. S23, indicating that the bioluminescent response doubles with addition of 117 (102-137, SE) µM DPD. Note the logarithmic response axis.

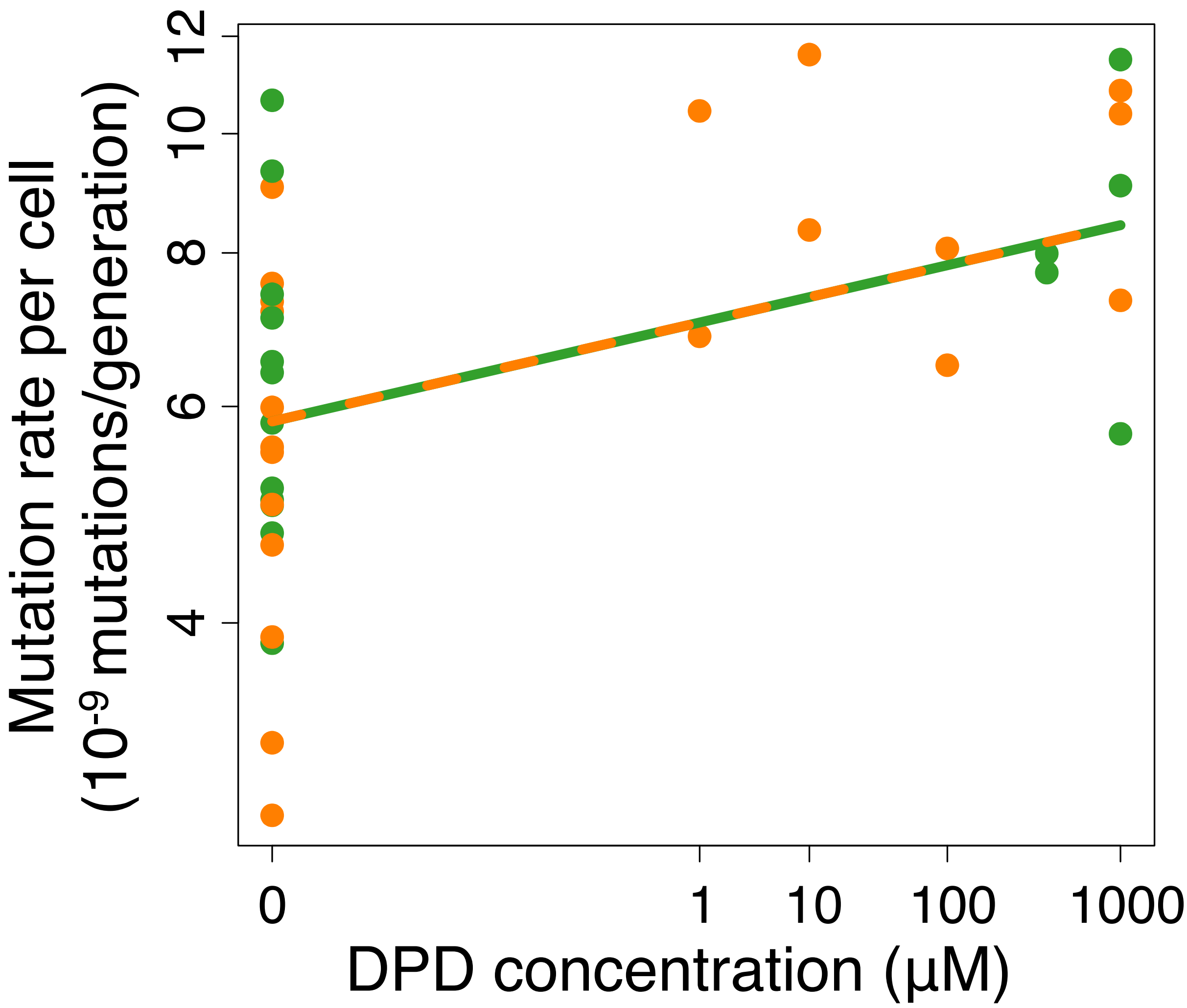

Supplementary Figure 6 | Effect of synthetic DPD on mutation rate to rifampicin resistance in wild-type *E. coli* K-12 (green) and otherwise isogenic Δ*luxS* (KX1228; orange). The line applies to both strains and is the fit from Model 5 (see Methods). All the cultures were grown in 100 mg/ml glucose, some of the points without DPD are shared with Fig. 2a. Note the transformed *x*-axis. For more information see Model 5 (in Methods) and main text.

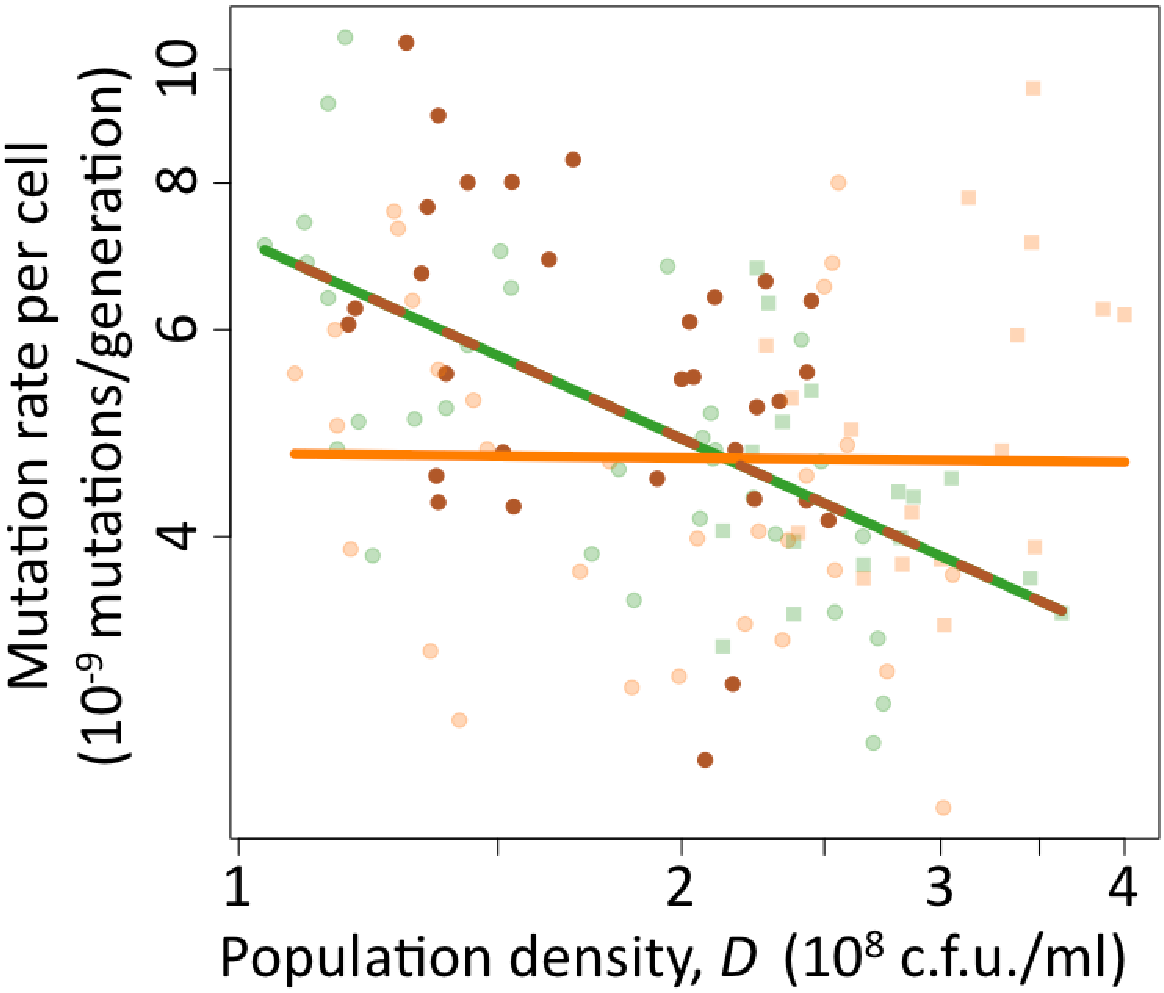

Supplementary Figure 7 | Mutation rate to rifampicin resistance in a Δ*lsrK* mutant. Mutation rate in the KX1228 Δ*lsrK* mutant (brown points) was assayed as in Fig. 2a. The data from Fig. 2a are shown semi-transparently, colours and shapes as given in that figure. The model shown in Fig. 2a (Model 3 in Methods) was re-fitted to include the Δ*lsrK* mutant data. This showed no significant difference between the wild-type and the Δ*lsrK* mutant, therefore these strains were combined, to give the fits shown by the lines here, which are very similar to those in Fig. 2a and Model 3.

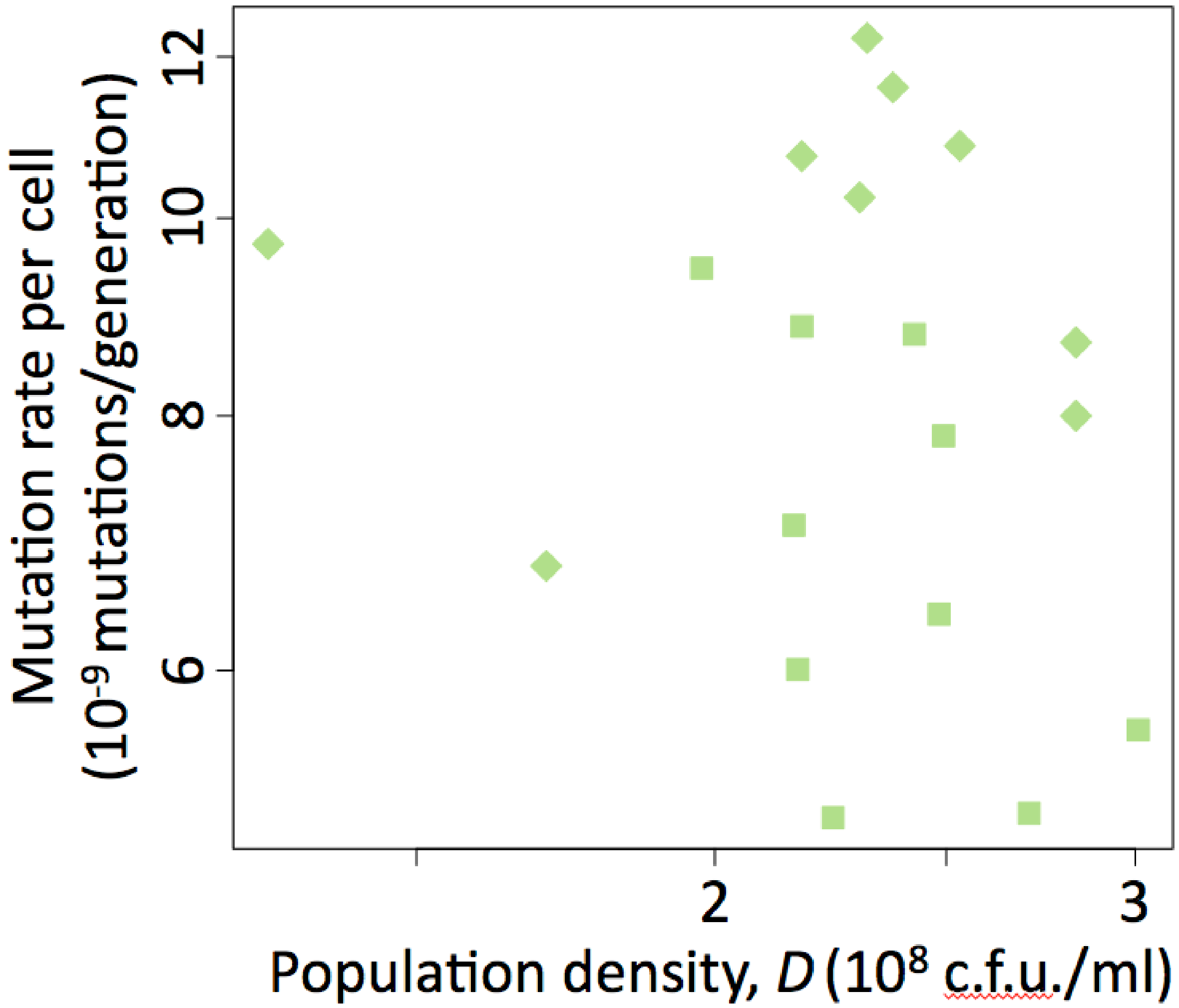

Supplementary Figure 8 | Mutation rate to rifampicin resistance in relationship to the final population density (*D*) in *E. coli* K-12 co-cultures. Chloramphenicol marked K-12 cells (KX1102) were co-cultured with either unmarked wild-type (squares) or Δ*luxS* mutant cells (diamonds). While there is a significant effect of co-cultured strain (Fig. 3) there is no significant effect of population density (see Model 8 in Methods).

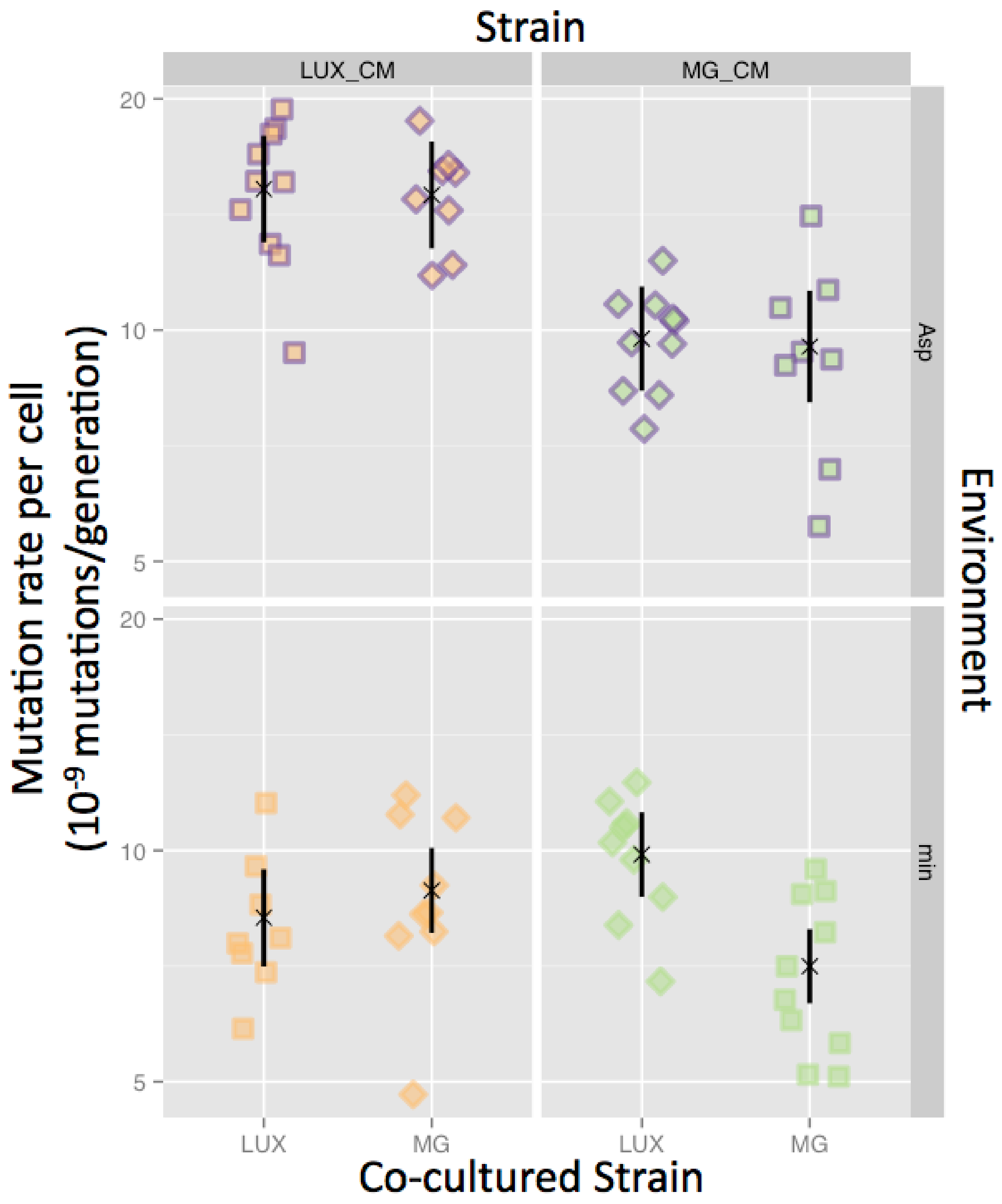

Supplementary Figure 9 | Mutation rate to Rif^R in chloramphenicol resistant *E. coli* K-12 strains either containing a Δ*luxS* mutation (LUX_CM, orange) or wild-type (MG_CM, green) co-cultured with either unmarked wild-type (MG) or Δ*luxS* mutant (LUX) cells in either aspartate containing (Asp, purple outline) or minimal (min) media. Squares indicate co-cultured strains with the same *luxS* genotype, diamonds with different *luxS* genotypes. Data points are randomly jittered left or right for clarity. The left half is the same data as is shown in Fig. 2b, the lower right panel is the same data as is shown in Fig. 3. Crosses and lines are fitted values and their 95% confidence intervals from Model 8 (see Methods), which analyses all the data simultaneously.

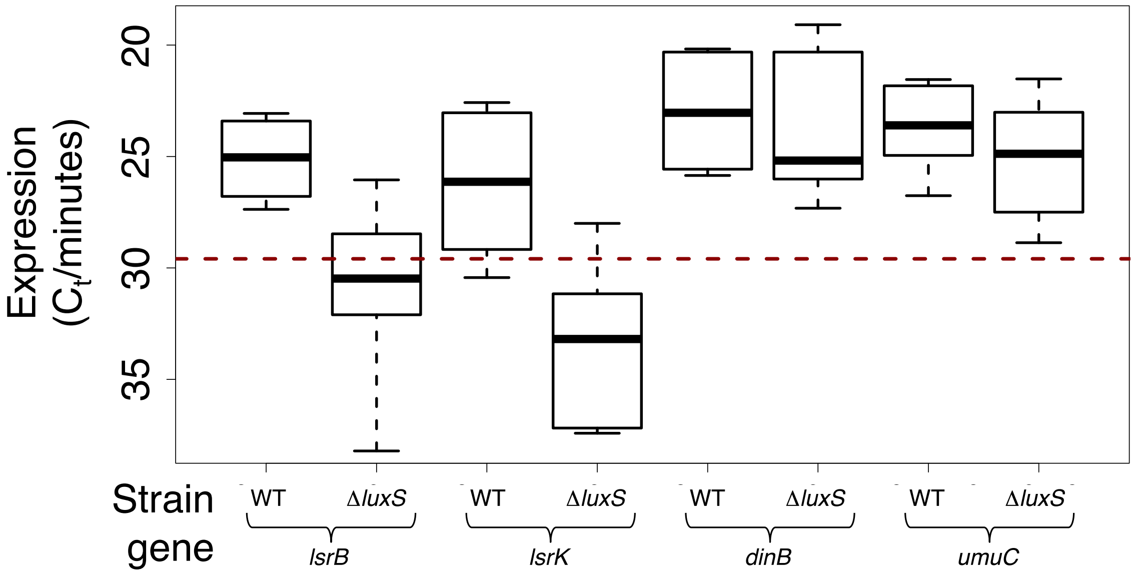

Supplementary Figure 10 | Expression of selected genes in the wild-type and Δ*luxS* strains measured by quantitative reverse-transcriptase polymerase chain reaction. Values are given on a C_t scale (time to reach threshold amplification in minutes). Bars indicate the median value, boxes the inter-quartile range and whiskers the maximum and minimum values recorded. The dashed red line indicates the limit of detection (median value of controls without the reverse-transcriptase step). These data are analysed in Model 10 (see Methods), with details in Supplementary Table S10 and Supplementary Fig. S24.

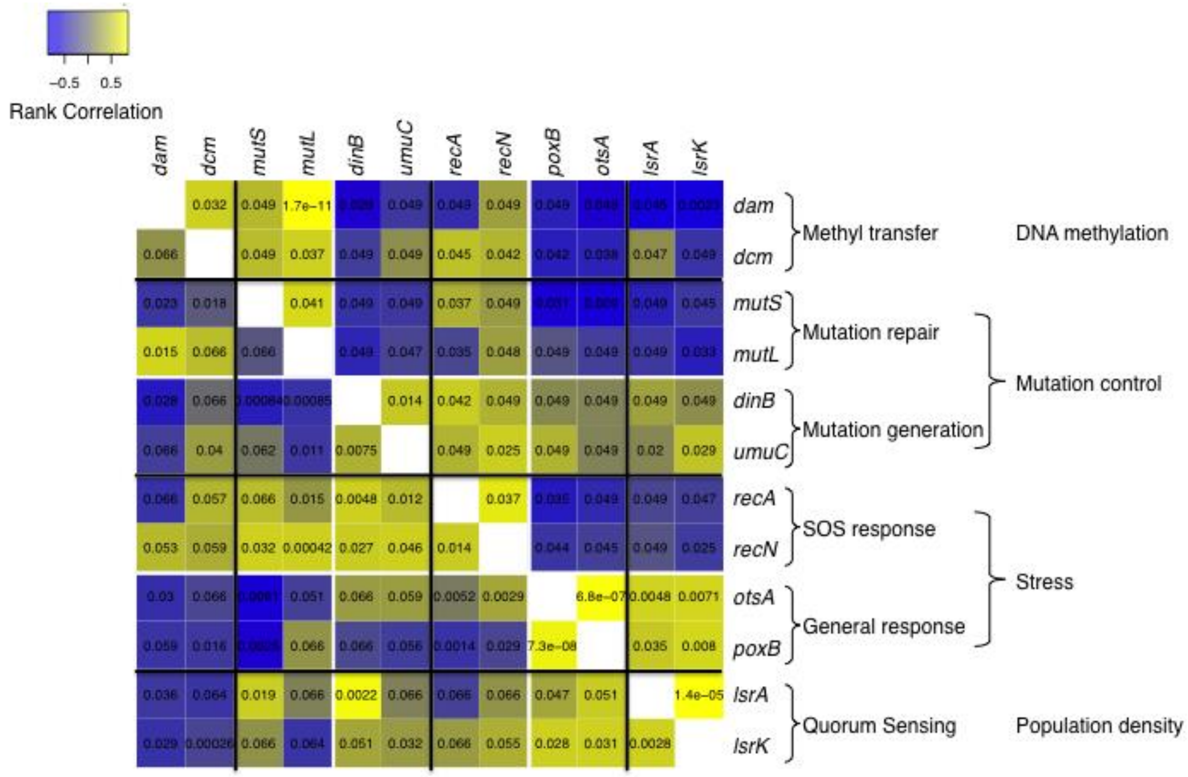

Supplementary Figure 11 | Correlations between Population density and stress, mutation control and DNA methylation in published *E. coli* gene expression data. Spearman rank correlations between the expression of two genes are shown by colour, either full correlations (above the diagonal) or partial correlations (below the diagonal), controlled for correlations with genes in other the other groups (i.e. DNA methylation, Mutation control, Stress or Population density). Correlation *P*-values (corrected for multiple testing, Hommel approach[1]) are given over the boxes. All values are weighted medians across 96 separate studies (see Methods). It is possible that two genes may be strongly positively associated in some studies but strongly negatively associated in others. In such cases the weighted median correlation may be close to zero but the weighted median *P*-value very low (as seen in the partial correlation between *mutL* and *dinB*). The number of samples used by different studies is highly skewed (median=11 but maximum=261). If many small studies contain a correlation in one direction, but the larger studies do not, there can be a high median correlation with a relatively high *P*-value (as seen in the full correlation between *lsrA* and *dam*).

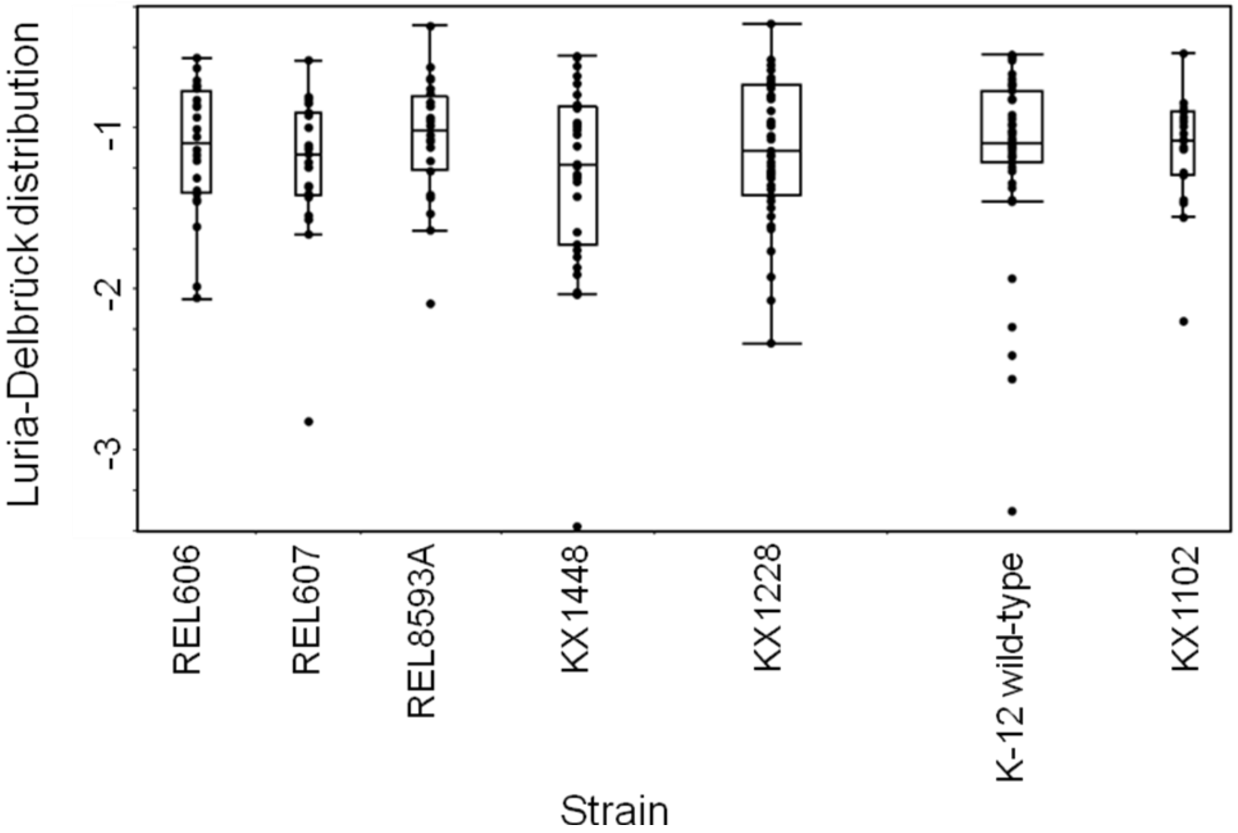

Supplementary Figure 12 | Slope values for the Luria-Delbrück curves in *Escherichia coli* strains. Luria-Delbrück curve is calculated as log(P_r) against log(r) (leaving out r=0), where P_r is the proportion of cultures that contain r or more mutants. Boxes correspond to inter-quartile ranges with a heavy bar at the median. Whiskers indicate the range excluding an outlier. Strains from left to right; N = 24, N = 21, N = 28, N = 31, N = 47, N = 50, N = 19, respectively.

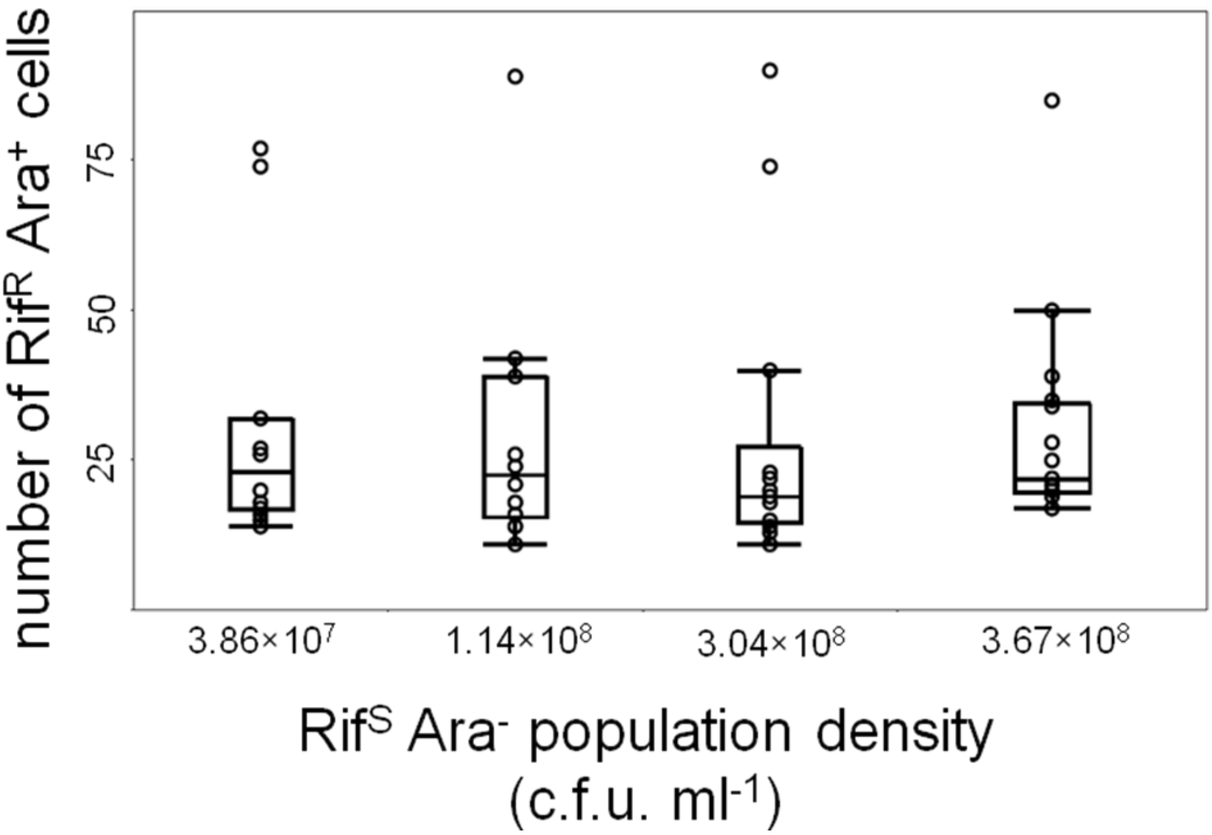

Supplementary Figure 13 | Plating efficiency of pre-existing rifampicin resistant (Rif^R) mutants at different densities of rifampicin sensitive (Rif^S) cells. Boxes correspond to inter-quartile ranges with a heavy bar at the median. Whiskers indicate the range excluding an outlier. We performed a fluctuation test with REL606 (Rif^S Ara$^-$) using 25, 100, 250 and 1000 mg/l of glucose yielding four different population densities, 3.86×10^7 (*N* = 14), 1.14×10^8 (*N* = 14), 3.04×10^8 (*N* = 14) and 3.67×10^8 (*N* = 17), respectively. Before plating of the entire Ara$^-$ culture on the selective tetrazolium agar medium, we inoculated each Ara$^-$ culture with around 25 pre-isolated REL607 (Rif^R Ara$^+$) cells.

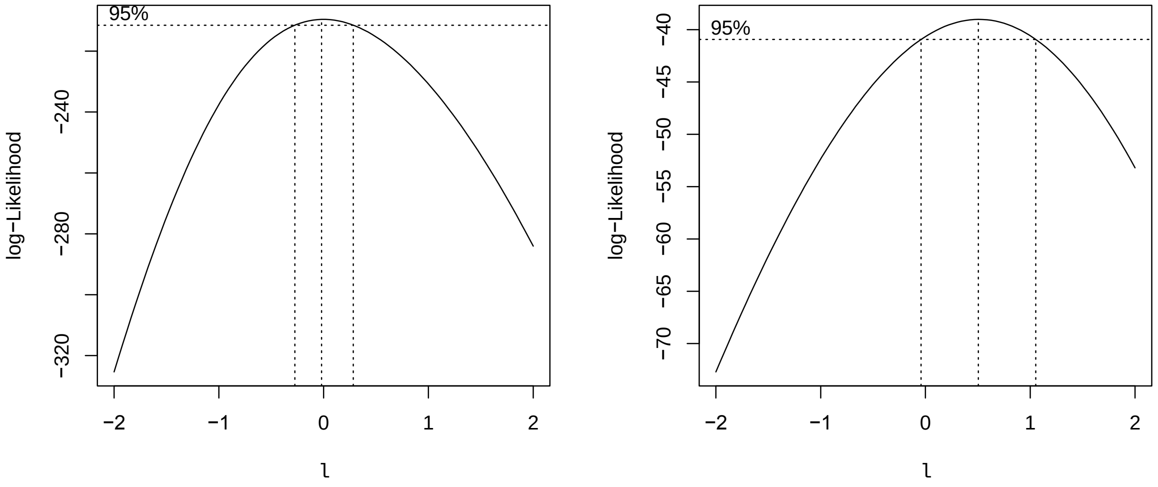

Supplementary Figure 14 | Box-Cox power transformations. Example likelihood curves for models with the same main effects as Models 3 and 8 (see Methods). The dotted lines give approximate 95% confidence intervals on the maximum likelihood transformation. λ is the power in a power transformation of the mutation rate, i.e. a value of 1 uses untransformed mutation rate. λ = 0 corresponds to a log transform. Other models of mutation rate gave similar curves all centred below 1 and mostly including zero.

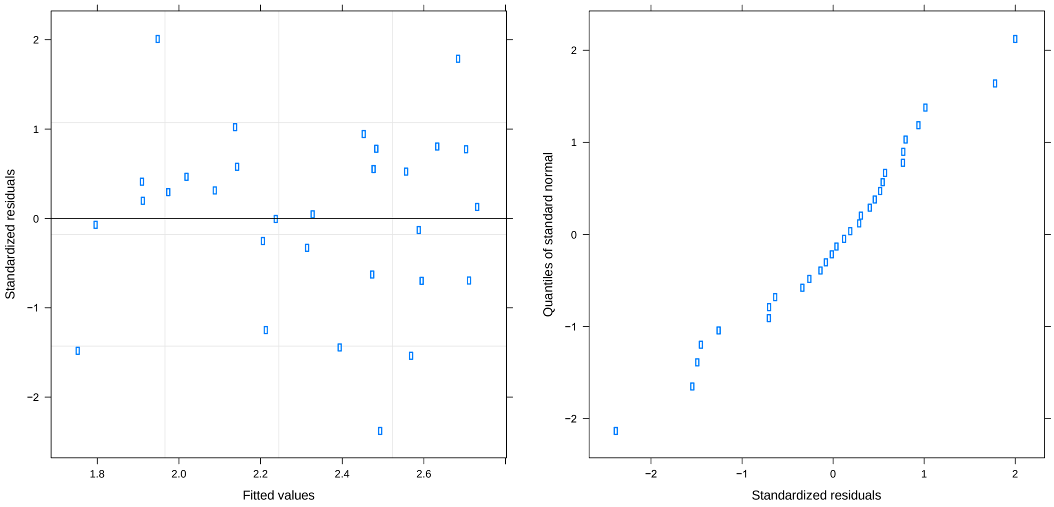

Supplementary Figure 15 | Diagnostic plots for Model 1. Standardised residuals by fitted values and normal quantile-quantile plot of standardised residuals

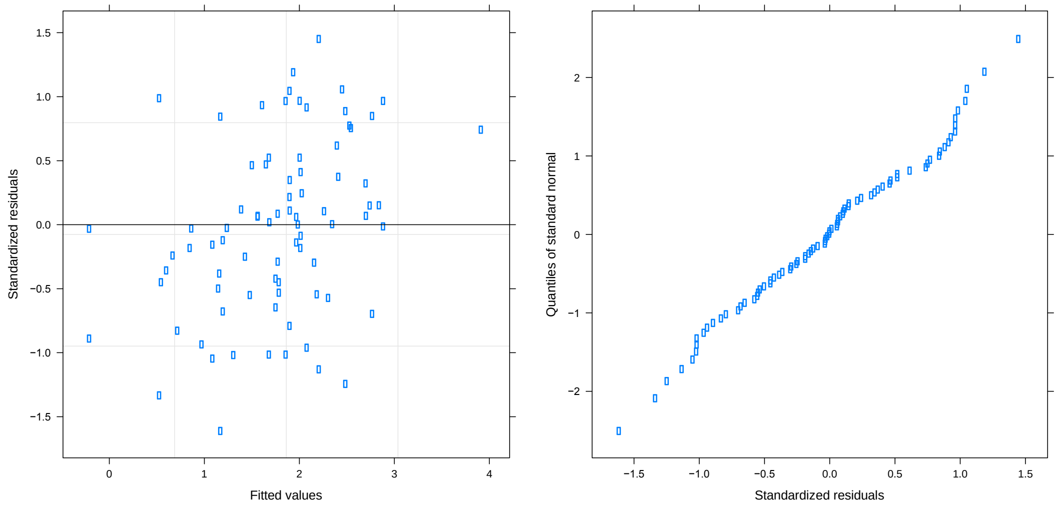

Supplementary Figure 16 | Diagnostic plots for Model 2. Standardised residuals by fitted values and normal quantile-quantile plot of standardised residuals.

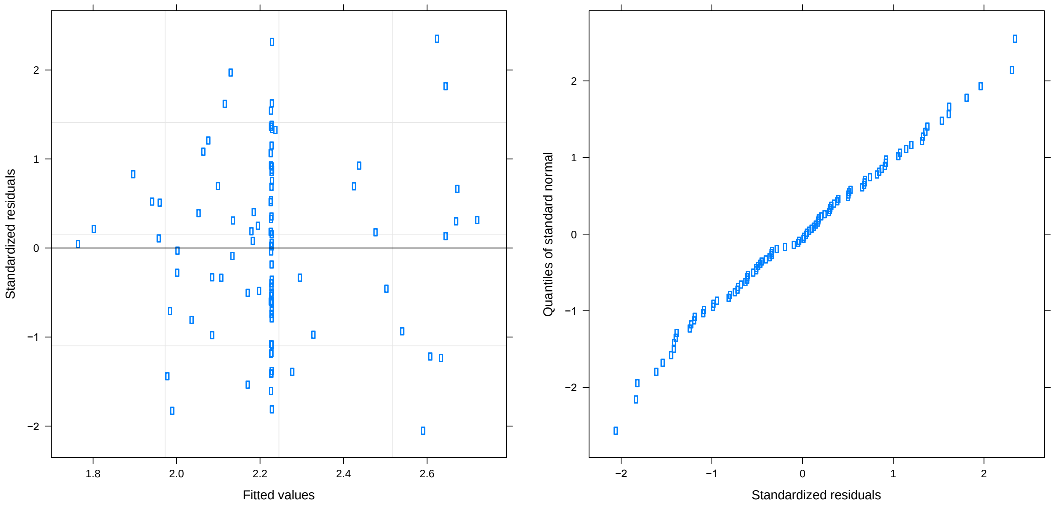

Supplementary Figure 17 | Diagnostic plots for Model 3. Standardised residuals by fitted values and normal quantile-quantile plot of standardised residuals.

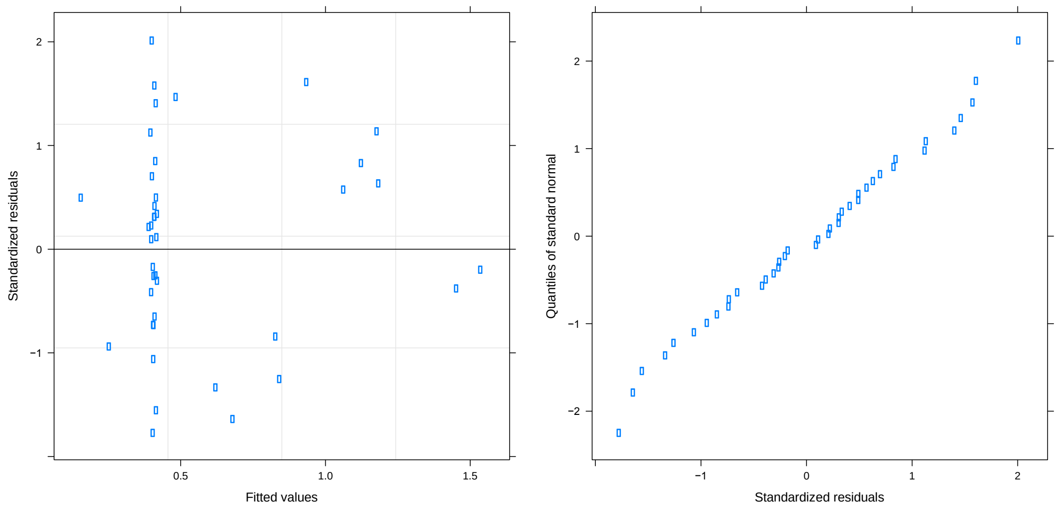

Supplementary Figure 18 | Diagnostic plots for Model 4. Standardised residuals by fitted values and normal quantile-quantile plot of standardised residuals.

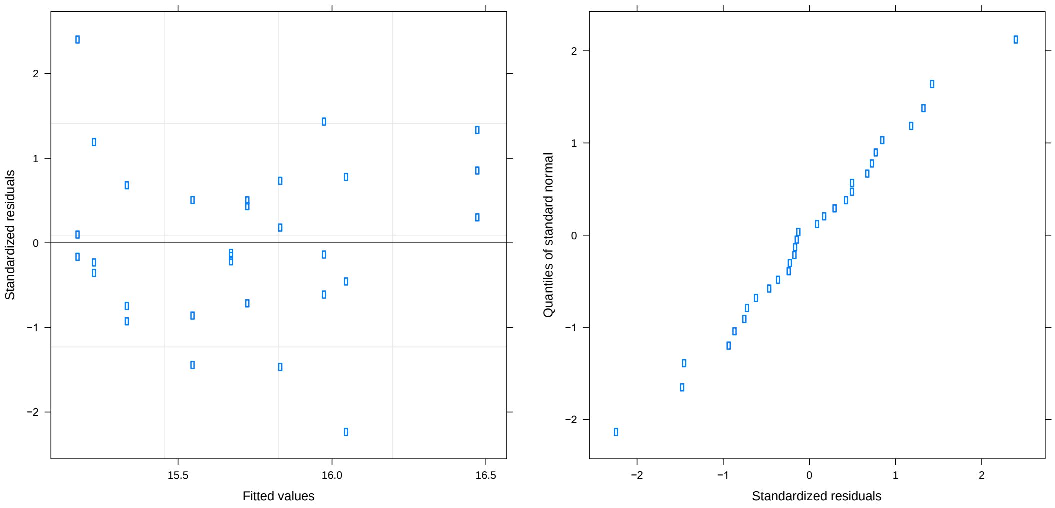

Supplementary Figure 19 | Diagnostic plots for Model 5. Standardized residuals by fitted values and normal quantile-quantile plot of standardized residuals.

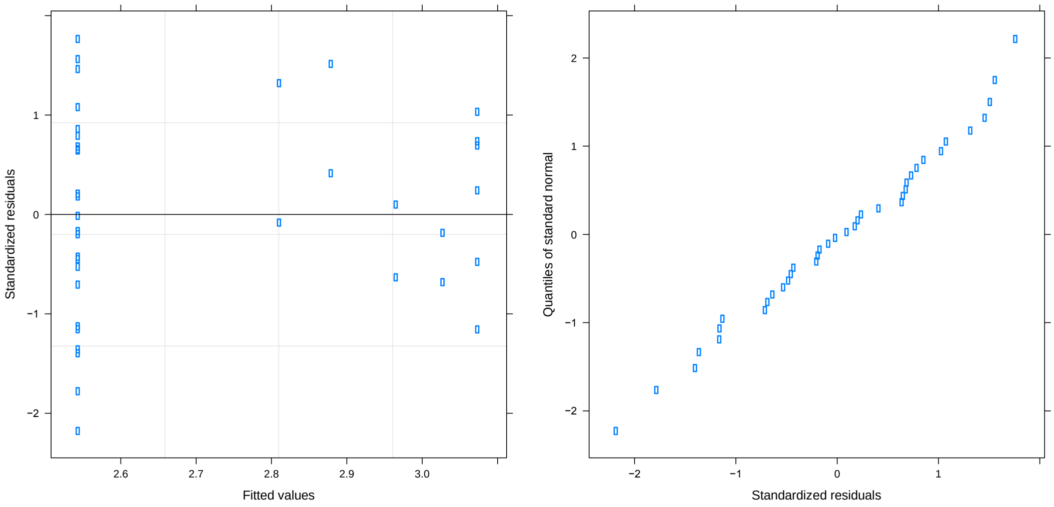

Supplementary Figure 20 | Diagnostic plots for Model 6. Standardised residuals by fitted values and normal quantile-quantile plot of standardised residuals.

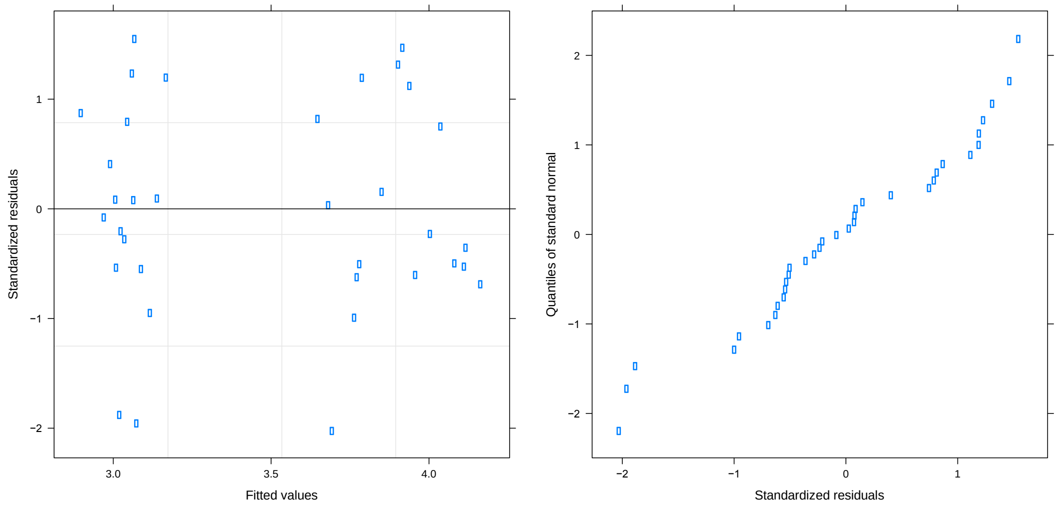

Supplementary Figure 21 | Diagnostic plots for Model 7. Standardised residuals by fitted values and normal quantile-quantile plot of standardised residuals.

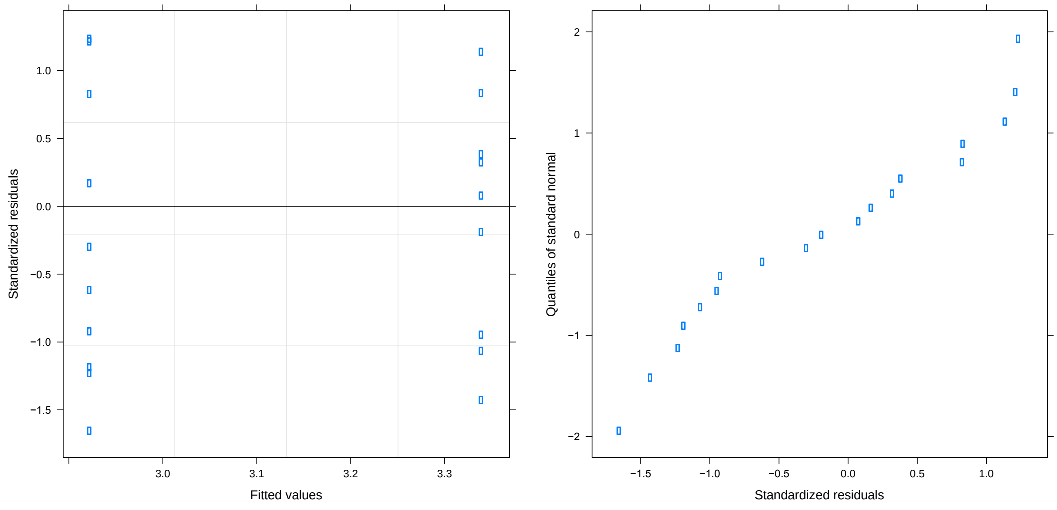

Supplementary Figure 22 | Diagnostic plots for Model 8. Standardised residuals by fitted values and normal quantile-quantile plot of standardised residuals.

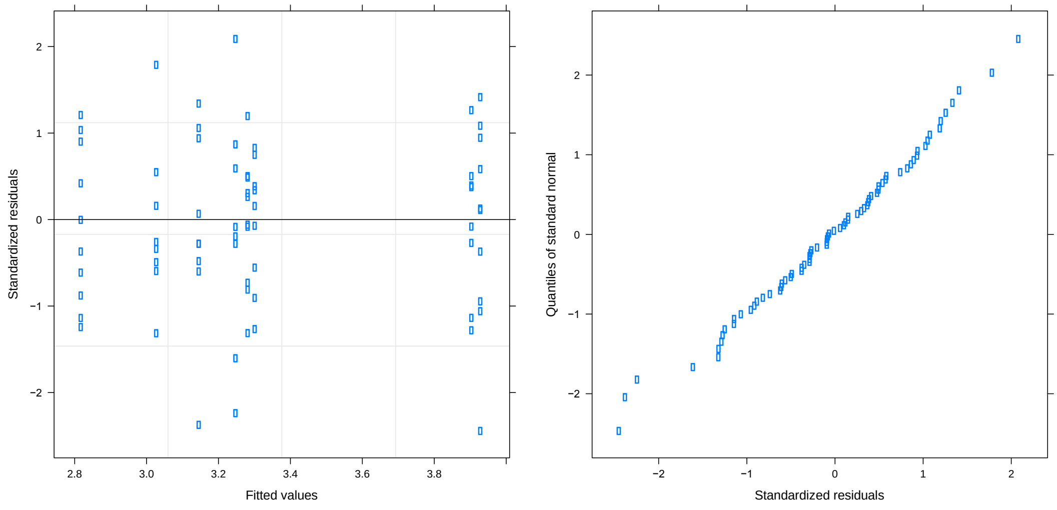

Supplementary Figure 23 | Diagnostic plots for Model 9. Standardised residuals by fitted values and normal quantile-quantile plot of standardised residuals.

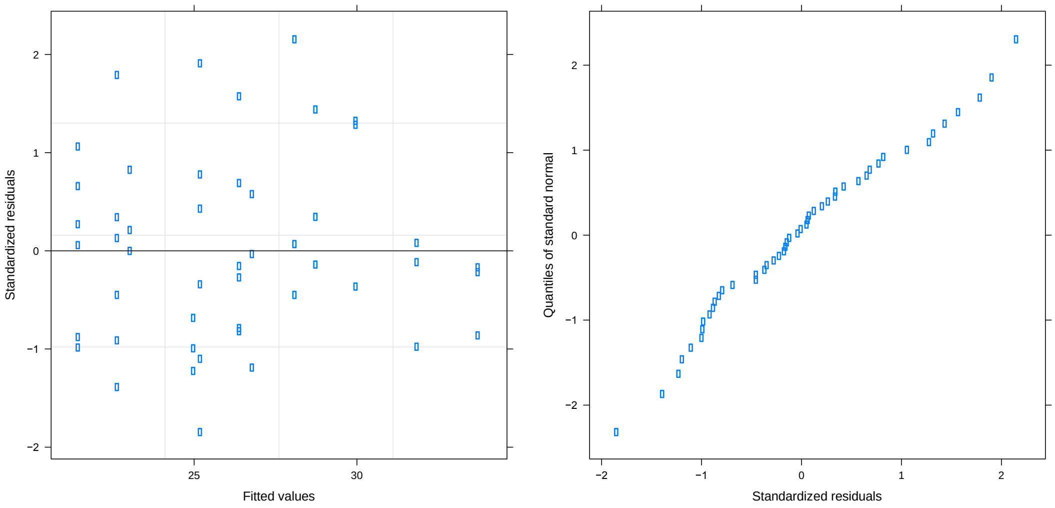

Supplementary Figure 24 | Diagnostic plots for Model 10. Standardized residuals by fitted values and normal quantile-quantile plot of standardized residuals.

	Degrees of freedom	Value	SE	*F*	*P*
Intercept	1	7.9	0.95	1853	0
w_{abs}	1	–0.39	0.066	35	2.4×10^{-6}

Supplementary Table 1 | ANOVA Table and fitted values for Model 1. See Methods for details.

	Degrees of freedom	Value	SE	*F*	*P*
Intercept	1	15	3.6	129	1.5×10^{-14}
$\log_2(D_{Av})$	1	–0.47	0.13	14	6.0×10^{-4}

Supplementary Table 2 | ANOVA Table and fitted values for Model 2. See Methods for details.

	Degrees of freedom	Value	SE	*F*	*P*
Intercept (Δ*luxS*)	1	2.2	0.067	3560	0
$\log_2(D)_{centred}$	1	0.0019	0.12	23	6.9×10^{-6}
Strain	1	–0.085	0.083	0.25	0.62
Strain : $\log_2(D_{centred})$	1	–0.53	0.15	12	7.3×10^{-4}

Supplementary Table 3 | ANOVA Table and fitted values for Model 3. See Methods for details.

	Degrees of freedom	Value	SE	*F*	*P*
Intercept (wild-type)	1	0.41	0.043	112	1.4×10^{-12}
$\log_2(D_{centred})$	1	–0.012	0.085	3.8	0.058
StrainPair	1	0.60	0.18	2.8	0.11
StrainPair : $\log_2(D_{centred})$	1	1.7	0.38	20	6.7×10^{-5}

Supplementary Table 4 | ANOVA Table and fitted values for Model 4. See Methods for details.

	Degrees of freedom	Value	SE	*F*	*P*
Intercept (BatchA)	1	16	0.13	258665	0
[DPD]	1	0.0085	0.0012	50	1.4×10^{-7}
Batch	1	-0.50	0.12	16	4.5×10^{-4}

Supplementary Table 5. ANOVA Table and fitted values for Model 5. See Methods for details.

	Degrees of freedom	Value	SE	*F*	*P*
Intercept	1	2.8	0.052	3018	0
Transformed([DPD])	1	0.027	0.0057	22	4.0×10^{-5}

Supplementary Table 6 | ANOVA Table and fitted values for Model 6. See Methods for details.

	Degrees of freedom	Value	SE	*F*	*P*
Intercept	1	3.1	0.078	7299	0
$\log_2(D)$	1	0.23	0.26	20	9.5×10^{-5}
Asp	1	0.96	0.1	79	4.9×10^{-10}
Asp : $\log_2(D)$	1	-1.3	0.44	8.2	0.0074

Supplementary Table 7 | ANOVA Table and fitted values for Model 7. See Methods for details.

	Degrees of freedom	Value	SE	*F*	*P*
Intercept (wild-type)	1	2.9	0.092	2679	0
Co-cultured strain	1	0.42	0.12	12	0.0034

Supplementary Table 8 | ANOVA Table and fitted values for Model 8. See Methods for details.

	Degrees of freedom	Value	SE	*F*	*P*
Intercept	1	2.8	0.12	9126	0
Strain (Δ*luxS*)	1	0.33	0.16	39	4.0×10^{-8}
Competitor (Δ*luxS*)	1	0.48	0.16	3.5	0.066
Asp	1	0.43	0.15	50	1.4×10^{-9}
Strain:Competitor	1	-0.60	0.23	1.9	0.17
Strain:Asp	1	0.33	0.21	18	6.4×10^{-5}
Competitor:Asp	1	-0.45	0.22	0.89	0.35
Strain:Competitor:Asp	1	0.59	0.30	4.0	0.050

Supplementary Table 9 | ANOVA Table and fitted values for Model 9. See Methods for details.

	Degrees of freedom	Value	SE	*F*	*P*
Intercept (wild-type *dinB/umuC* BlockA)	1	25	0.31	15928	0
Gene (*lsrB*)	2	1.6	0.55	26	5.4×10^{-8}
(*lsrK*)		3.5	0.64		
Strain	1	1.2	0.64	17	1.6×10^{-4}
Block	1	-3.7	0.45	68	3.1×10^{-10}
Gene:Strain (*lsrB*)	2	3.9	1.5	4.9	0.012
(*lsrK*)		3.8	1.5		

Supplementary Table 10. ANOVA Table and fitted values for Model 10. See Methods for details.

**Model**	**Slope of log(mutation rate) against log(density) ±SE and *P* value for test that it equals zero**
Full model	–0.62±0.13 P=3.6x10^{-5}
Averaged *D*	–0.53±0.12 P=2.1 x10^{-4}
Reversed (Averaged μ)	–0.82±0.11 P=3.3 x10^{-5}

Supplementary Table 11 | Estimates of the effect of density on mutation rate under different modelling assumptions. All models relate $\log_2$ mutation rate (μ $cell^{-1}$ $generation^{-1}$) to $\log_2$ density (D colony forming units ml^{-1}) for the data plotted in Fig. 1. See Supplementary Note 1 for details.

Supplementary Note 1

Testing modelling assumptions.

(A) Fluctuation Tests. There are many technical factors in fluctuation tests, as used here for assaying mutation rates, which in principle could give spurious relationships[2]. For instance, while the assumption of a fluctuation test is that the mutations identified via a selective environment (here the presence of rifampicin) have no fitness effect in the environment in which they arose, there often *are* fitness effects of antibiotic resistance[3], potentially leading to biases mutation rate estimates. In the case of rifampicin resistance, that cost of resistance varies among environments with the demand for RNA polymerase[4]. Therefore, IF that demand varied with population density in an appropriate direction, it might provide a spurious explanation for the results in Fig. 1. We refute this and other possible spurious explanations for these relationships by subsequent experiments:

Any spurious explanations based on interactions between the environment and technical features of the fluctuation test cannot explain results based on the difference between two strains co-cultured in the same environment (Supplementary Fig. S4). In addition we test multiple independent manipulations (manipulation of density, Fig. 1; manipulation of genotype Fig. 2a; and manipulation physical, Fig. 2b and social environment, Fig. 3) each of which shows a difference and each of which would require its own correlation with a technical effect if our results were to have a spurious explanation.

In addition, there are specific assumptions of the fluctuation test that we assessed directly. Firstly we calculated the slope of the Luria-Delbrück curve for mutation rate estimates in each strain (Supplementary Fig. S11). A perfect Luria-Delbrück distribution has a slope of –1 and distortions of this curve might tell us about possible deviations from this distribution. Although the number of mutants in our system does not have a perfect Luria-Delbrück distribution, which is something expected for biological systems, the distribution stays the same in all used strains (Supplementary Fig. S12). We also find that the slope values are not correlated with population density, mutation rates or with any other parameter used in statistical models. Secondly we tested plating efficiency of rifampicin resistant (Rif^R) cells at different densities. We performed a fluctuation test with REL606 (Rif^S Ara^-), as described in Methods, using 25, 100, 250 and 1000 mg/l of glucose yielding four different population densities. However, before plating of the entire Ara^- culture on the selective tetrazolium agar medium, we inoculated each Ara^- culture with around 25 pre-isolated REL607 (Rif^R Ara^+) cells. We observed that Rif^R cells present in the culture before plating makes a colony regardless of the plated population density (Supplementary Fig. S13).

(B) Linear models. The linear modelling in the analyses here makes the assumption that there is no error in the explanatory variables. For the continuous variables used, such as population density, this assumption will never be completely correct. We therefore tested the influence of this in modelling our data. For regressing mutation rate (μ) against population density (D), cell counts in a non-selective environment contribute to the numerator of the explanatory variable and the denominator of the response variable. Therefore in this case we have a specific expectation for the effect of deviation from the assumption of error-free explanatory variables: more error in these counts will give a more negative (or less positive) dependence.

The degree of this error may be manipulated by averaging more or less data. This we did for the data shown in Fig. 1a, where the experimentally altered variable was nutrient (glucose) concentration ([*glc*]). A full analysis gives a dependence of –0.62±0.13 (Supplementary Table S11). Averaging the value of *D* for each value of [*glc*] will reduce any error in *D*. Re-fitting with averaged (median) *D* values does indeed give a less negative dependence consistent with some effect of error in the explanatory variable. Nonetheless, both estimates are significantly negative and well within each others' error bounds (Supplementary Table S11). It is also possible to increase the error by reversing the regression, treating the mutation rate as the explanatory variable (the number of mutational events, *m*, contributes to this value and has a substantial error estimated in the course of its maximum likelihood calculation from mutant counts). As expected, this shifts the slope in the opposite direction and the absolute shift is much greater than the effect of manipulating the error in *D* by averaging (Supplementary Table S11). Nonetheless, the standard errors of all three estimates overlap. We therefore conclude that noise in our explanatory variables, specifically our estimates of *D*, is of little importance for our findings.

The small effect identified above could nonetheless have an influence when choosing between explanatory variables which may experience the effect to different degrees. This is done explicitly in Model 2 (see Methods). Therefore, for this model, we averaged *D* and other estimated explanatory variables within each strain-environment combination (where environment includes nutrient, competitor strain, culture volume and growth period) in this analyses. Again the dependence on density is less negative than obtained from a naïve analysis (as is apparent from Fig. 1b), however, the relationship of mutation rate with population density remains highly significant (Model 2 in Methods, Supplementary Table S2). It is important to note that this approach is, in general, unnecessarily conservative and therefore only taken in Model 2 – variation in population density within strain-environment treatments is meaningful in these experiments. This is demonstrated in Fig. 2b and Supplementary Fig. S7, where, in each case, *all* the variation in density for the K-12 strains is within a single glucose, volume and incubation time treatment (250 mg/ml Glucose 1ml cultures incubated for 24h). Nonetheless, in each case, there is a relationship with density in one treatment (aspartate in Fig. 2b, Δ*luxS* in Supplementary Fig. S4) but not the other.

Supplementary Note 2

In our data, to explain the effect of social environment seen in Fig. 3 by stress-induced mutagenesis would require the secretion of a stressful or mutagenic compound by the Δ*luxS* mutant and/or a reduction its depletion of stress-relieving or anti-mutagenic nutrients. This effect of Δ*luxS* on the social environment is complemented (i.e. the difference between a wild-type and a Δ*luxS* environment is removed) by addition of aspartate (Supplementary Fig. S9). If the underlying mechanism involved stress-induced mutagenesis, we would infer that aspartate is relieving the secretion of a stressful or mutagenic compound by the *luxS* mutant and/or a reduction its depletion of stress-relieving or anti-mutagenic nutrients. We would therefore expect that the effect of aspartate on the Δ*luxS* mutant alone would be a reduction in mutation rate. Contrary to this we find a general and significant *increase* in the mutation rate with the addition of aspartate (Supplementary Fig. S9 and Model 9 in Methods). Again, this suggests an independence of the MRP identified here and stress-induced mutagenesis.

Supplementary Methods

#This script reproduces the analysis and figures 1-3 from the main text of the paper (Krasovec et al. 2014).

#It is designed to be run using R (see r-project.org)

#Reading in the data requires setting the working directory appropriately e.g.:

#setwd("~/folder_containing_data")

#(remove the comment sign (#) in the line above and insert an appropriate file path before use)

#This script may either be run in one go, simply to reproduce the figures, by copying and pasting this section (from #This script to the date at the end) into a .R file, saving that file (e.g. calling it KrasovecCode.R) in the same folder as the data and using the following command in R (again remove comment before use):

#source("KrasovecCode.R")

#Equally, this script may be worked through line by line to look at the models in more detail.

#In either case it requires the KrasovecData worksheet of the associated data file exported from Excel as a comma separated value file, KrasovecData.csv, which can be read in:

```
d <- read.csv("KrasovecData.csv", header=TRUE)
```

#the nlme package is required for the linear models and the RColorBrewer package for the colours used in the figures:

```
require(nlme)

require(RColorBrewer)
```

#Each Figure and analysis uses a subset of the data based on the 'Figure' column. Some of the data is common between figures 1a and 2a, meaning that some rows are duplicated apart from their value in the 'Figure' column. This is made clear by the 'ID' column which is unique for each unique row.

#In each case a model is generated called 'mod' which, if desired, may be inspected using commands such as:

```
#summary(mod)

#intervals(mod)

#anova(mod)

#The diagnostic plots given in supplementary figures may be regenerated in each case using:

#plot(mod)

#qqnorm(mod)


#Figure 1a


d1a <- d[d$Figure == "Fig1a", ]


#The model used (Model 1)

mod <- gls(log2(Mutation_rate_per1e9)~Wabs, data=d1a, weights=varPower(form=~Nt_plates))


#Choose appropriate colours and plot characters

pch <- 19

pal <- brewer.pal(8, "Paired")

cols <- pal[4]


#Create the plot

pdf("Fig1a.pdf")

plot(Mutation_rate_per1e9~Wabs, data=d1a, log="y", ylim=c(2.5,11), main="Mutation Rate versus Absolute Fitness", type="n", xlab="Wabs", ylab="Mutation rate x 10^9")

points(Mutation_rate_per1e9~Wabs, data=d1a, pch=pch, col=cols,cex=2)

lines(d1a$Wabs, 2^fitted(mod), lwd=6)
```

```
dev.off()

#Figure 1b

d1b <- d[d$Figure == "Fig1b", ]

#The model used (model 2):
mod <- lme(log2(Mutation_rate_per1e9)~log2(density_av), random= ~1|set/Experiment, data=d1b, weights=varPower(form=~C_plates))

#Choose appropriate colours and plot characters
pal <- brewer.pal(8, "Paired")
cols <- rep(pal[1], nrow(d1b))
cols[d1b$Strain == "0_minus"] <- pal[2]
cols[d1b$Strain == "20_minus"] <- pal[6]
pch <- rep(15, nrow(d))
pch[d1b$Competitor == "none"] <- 19

#create a subset of data containing the co-cultures with mutation rates for both strains only
d1b_co <- d1b[d1b$Experiment %in% unique(d1b$Experiment)[table(d1b$Experiment)==2],]

#Create the plot
pdf("Fig1b.pdf")
plot(Mutation_rate_per1e9~density_per_ml, data=d1b, log="xy", main="Mutation Rate versus Density", type="n", xlab="Overall culture Density", ylab="Mutation rate x 10^9", ylim=c(0.6, 17))
```

```
segments(x0=d1b_co$density_per_ml[as.logical(1:30 %% 2)], x1=d1b_co$density_per_ml[as.logical(0:29 %% 2)], y0=d1b_co$Mutation_rate_per1e9[as.logical(1:30 %% 2)], y1=d1b_co$Mutation_rate_per1e9[as.logical(0:29 %% 2)] )

points(Mutation_rate_per1e9~density_per_ml, data=d1b, pch=pch, col=cols, cex=2)

lines(d1b$density_av[order(d1b$density_av)], 2^fitted(mod, level=0)[order(d1b$density_av)], lwd=6)

dev.off()



#Figure 2a



d2a <- d[d$Figure == "Fig2a", ]



#The model used (Model 3):

mod <- gls(log2(Mutation_rate_per1e9)~I(log2(density_per_ml)-log2(mean(density_per_ml)))*Strain, data=d2a, weights=varComb(varIdent(form=~1|Strain),varPower(form=~C_plates)))



#Choose appropriate colours and plot characters

pal <- brewer.pal(8, "Paired")

cols <- rep(pal[4], nrow(d2a))

cols[d2a$Strain == "LUX"] <- pal[8]

pch <- rep(15, nrow(d2a))

pch[d2a$Competitor == "none"] <- 19



#Create the plot

pdf("Figure2a.pdf")

plot(Mutation_rate_per1e9~density_per_ml, data=d2a, log="xy", main="Effect of luxS on mutation rate plasticity", type="n", xlab="cells/ml", ylab="Mutation rate x 10^9")
```

```
points(Mutation_rate_per1e9~density_per_ml, data=d2a, pch=pch, col=cols, cex=1.2)

 lines(c(max(d2a$density_per_ml[d2a$Strain == "MG"]), min(d2a$density_per_ml[d2a$Strain == "MG"])), c(2^min(predict(mod)[d2a$Strain == "MG"]), 2^max(predict(mod)[d2a$Strain == "MG"])), lwd=6, col=pal[4])

 lines(c(max(d2a$density_per_ml[d2a$Strain == "LUX"]), min(d2a$density_per_ml[d2a$Strain == "LUX"])), c(2^min(predict(mod)[d2a$Strain == "LUX"]), 2^max(predict(mod)[d2a$Strain == "LUX"])), lwd=6, col=pal[8])

dev.off()



#Figure 2b



d2b <- d[d$Figure == "Fig2b", ]



#The model used (Model 7):

mod<-gls(log2(Mutation_rate_per1e9)~I(log2(density_per_ml)-mean(log2(density_per_ml)))*aspEnv, data=d2b, weights=varPower(form=~Innoculum_size))



#Choose appropriate colours and plot characters

pal<-brewer.pal(12,"Paired")

bgcols<-rep(pal[7], nrow(d2b))

ecols<-rep(pal[7], nrow(d2b))

ecols[d2b$aspEnv=="Asp"]<-pal[10]

pch <- rep(22, nrow(d))

pch[d2b$Competitor=="MG"]<-23



pdf("Figure2b.pdf")

plot(Mutation_rate_per1e9~density_per_ml, data=d2b, log="xy", main="Effect of Aspartate on luxS mutatant plasticity", type="n", xlab="cells/ml", ylab="Mutation rate x 10^9")
```

points(Mutation_rate_per1e9~density_per_ml, data=d2b, pch=pch, col=ecols,bg=bgcols, cex=2, lwd=3)

lines(c(max(d2b$density_per_ml[d2b$aspEnv=="Asp"]),min(d2b$density_per_ml[d2b$aspEnv=="Asp"])), c(2^min(predict(mod)[d2b$aspEnv=="Asp"]),2^max(predict(mod)[d2b$aspEnv=="Asp"])), lwd=6, col=pal[10])

lines(c(min(d2b$density_per_ml[d2b$aspEnv=="min"]),max(d2b$density_per_ml[d2b$aspEnv=="min"])), c(2^min(predict(mod)[d2b$aspEnv=="min"]),2^max(predict(mod)[d2b$aspEnv=="min"])), lwd=6, col=pal[7])

dev.off()

#Figure 3

d3 <- d[d$Figure == "Fig3",]

#ensure appropriate factor levels:

d3$Competitor <- factor(d3$Competitor)

#Create the plot

pdf("Figure3.pdf")

plot(Mutation_rate_per1e9~Competitor, data=d3, log="y", main="Effect of social context", xlab="Competitor", ylab="Mutation rate x 10^9", ylim=c(4.8, 12.5))

dev.off()

#17th March 2014

Supplementary References

1. Hommel, G. A Stagewise rejective multiple test procedure based on a modified Bonferroni Test. *Biometrika* **75**, 383–386 (1988).

2. Pope, C. F., O'Sullivan, D. M., McHugh, T. D. & Gillespie, S. H. A practical guide to measuring mutation rates in antibiotic resistance. *Antimicrob. Agents Chemother.* **52,** 1209–1214 (2008).

3. Andersson, D. I. The biological cost of mutational antibiotic resistance: any practical conclusions? *Curr. Opin. Microbiol.* **9,** 461–465 (2006).

4. Hall, A. R., Iles, J. C. & MacLean, R. C. The fitness cost of rifampicin resistance in *Pseudomonas aeruginosa* depends on demand for RNA polymerase. *Genetics* **187,** 817–822 (2011).
